# YOLOv8n-DSLW: A Deployment-Oriented AI-Enabled Vision-Sensing Model for Tiny Strawberry Disease and Pest Detection in Greenhouse Images

**DOI:** 10.3390/s26154831

**Published:** 2026-07-30

**Authors:** Lanxin Chen, Guanjie Wang, Zhekai Cai, Zixiang Yi, Dongxu Zhang

**Affiliations:** 1 Ulster College, Shaanxi University of Science and Technology, Xi’an 710021, China; 202315010201@sust.edu.cn (L.C.); 202315020118@sust.edu.cn (G.W.); 202315010109@sust.edu.cn (Z.C.); 202415020222@sust.edu.cn (Z.Y.); 2 College of Mechanical and Electrical Engineering, Shaanxi University of Science and Technology, Xi’an 710021, China

**Keywords:** vision sensing, camera-based sensing, AI-enabled sensors, greenhouse monitoring, strawberry disease detection, tiny object detection, YOLOv8n, lightweight object detection, deployment-oriented detection

## Abstract

Camera-based visual sensing provides a non-destructive and scalable approach for monitoring strawberry diseases and pests in greenhouse environments. However, greenhouse images acquired under practical cultivation conditions often contain early-stage tiny lesions, complex leaf backgrounds, uneven target scales, illumination variations, and partial occlusions, making accurate and efficient visual detection challenging. To address these issues, this study proposes YOLOv8n-DSLW (YOLOv8n enhanced by Dense reuse, Shuffle attention, LSKA–LAMP lightweight modeling, and Wise-IoU optimization), an AI-enabled vision-sensing detection model based on YOLOv8n for tiny strawberry disease and pest detection. Specifically, Shrink Residual Dense Block (ShrinkRDB) dense connection blocks and the C2f with Shuffle Attention (C2fSA) module are introduced to preserve weak lesion textures and suppress background interference in greenhouse visual data. A high-resolution P2 detection layer combined with Wise-IoU (WioU) dynamic regression loss is further incorporated to enhance tiny-target perception and localization. In addition, the Spatial Pyramid Pooling-Fast with Large Separable Kernel Attention (SPPF-LSKA) module strengthens contextual modeling under occlusion and clutter, while Layer-Adaptive Magnitude-based Pruning (LAMP) is adopted to mitigate model redundancy and improve the accuracy–efficiency balance. Experiments on a self-collected greenhouse strawberry disease and pest dataset show that YOLOv8n-DSLW achieves a mean Average Precision at 0.5 IoU threshold (mAP@0.5) of 94.3% and a mAP@0.5:0.95 of 77.5%, outperforming the YOLOv8n baseline. The final model has a parameter count of 4.386 M and a computational cost of 27.6 GFLOPs, achieving a frame rate of 45 FPS on the test workstation. It shows application potential for real-time visual monitoring in greenhouses under controlled data acquisition conditions. The results demonstrate that the proposed method improves tiny lesion detection under dense targets, complex backgrounds, and leaf occlusions, providing an AI-enabled vision-sensing framework for automated strawberry health monitoring in greenhouses. Nevertheless, due to limitations associated with imaging equipment, dataset representativeness, and the inherent constraints of the algorithm, further optimization and validation are required to support large-scale field deployment.

## 1. Introduction

Strawberry, belonging to the Rosaceae family and originating from China, is one of the most widely cultivated economic crops worldwide due to its distinctive flavor and high vitamin C content [1]. Recent studies report that strawberry is cultivated on approximately 0.37–0.39 million hectares worldwide, with annual production exceeding 8–9 million tons; major production is concentrated in Asia, the Americas, and Europe [2,3]. Among them, China has become the world’s largest strawberry producer, with an annual production of approximately 4.2 million tons, accounting for more than 40% of global production. Owing to its short production cycle, high economic returns, and suitability for protected cultivation, strawberry production has become an important high-value horticultural sector for promoting industrial development and increasing farmers’ income in China [4,5].

In China, especially in northern regions, strawberry production is predominantly conducted under greenhouse or protected cultivation systems [5,6]. However, greenhouse microclimates characterized by relatively high temperature, high humidity, low light, and restricted air exchange can promote the development and spread of strawberry diseases, particularly gray mold, powdery mildew, and anthracnose [6,7,8,9]; aphids and two-spotted spider mites are also important herbivorous pests of strawberry [10]. Traditional manual inspection methods are labor-intensive, inefficient, and prone to misjudgment, making them incapable of meeting the requirements of large-scale cultivation for comprehensive greenhouse monitoring [10]. Although laboratory-based detection approaches, such as molecular biological analysis and hyperspectral imaging, can achieve relatively high accuracy, they are time-consuming, costly, and highly dependent on professional equipment and expertise, thereby limiting their large-scale application and failing to satisfy the practical demand for early detection and early prevention of strawberry diseases and pests [11].

In recent years, with the rapid development of computer vision and intelligent sensing technology, remarkable progress has been achieved in crop disease and pest detection. Traditional image-processing- and machine-learning-based methods, such as Random Forest (RF) and Naive Bayes (NB) [12], can recognize disease lesions to a certain extent; however, these methods rely heavily on manually designed features and exhibit poor robustness in complex greenhouse image acquisition environments. In deep learning, both Transformer architectures and convolutional neural networks (CNNs) are capable of automatically extracting deep semantic features from sensor-captured images [13], demonstrating strong robustness and enabling real-time object detection after appropriate optimization [14].

In the field of agricultural disease, pest, and crop target detection, Ai et al. developed the YOLO-SSM (YOLOv8 incorporating SSPDConv, ESPPFCSPC, and MPDIoU) algorithm based on the YOLOv8 architecture. By introducing the SPD-SimAM-Convolution (SSPDConv) module integrating Spatial-to-Depth (SPD) transformation and A Simple, Parameter-Free Attention Module (SimAM) three-dimensional attention, the proposed method effectively addressed the issue of tiny lesion feature loss and severe background interference in dense and complex environments, achieving a mAP of 89.7% [15]. Zheng et al. proposed the lightweight Aphid-YOLO model based on Mosaic9 data augmentation, tiny path aggregation network (TPANet) feature fusion, and the normalized Wasserstein distance (NWD) loss function, realizing real-time recognition and precise localization of extremely tiny aphids in complex farmland environments with a mAP@0.5 of 83.4% [16]. Wang et al. developed the TGL-YOLO algorithm based on an improved YOLO11 framework. By incorporating the Three-Scale Dynamic Block (TSDBlock), Gated Positional Spatial Transformer (GPST), and Large Spatial Pyramid Attention (LSPA), the model achieved precise multi-scale crop disease detection under complex natural environments, with a mAP@0.5 of 79.3% [17]. Chen et al. proposed an YOLO11-ARL lightweight framework based on YOLOv11n for early rare disease detection in long-tail agricultural scenarios, reducing the number of parameters by 51.93% [18]. Yuan et al. constructed a Hierarchical Attention-Driven Dense Fusion Network (HADF-Net) based on YOLOv8. Through dense aggregation and cross-layer shortcut connections, the network effectively addressed leaf occlusion in rice fields and achieved reliable tiny disease and pest detection [19]. Dai et al. introduced the lightweight YOLOv11-rdtnet model by embedding an Efficient Multi-scale Attention (EMA) mechanism and the Batch-Normalization-Scale Importance (BNScaleImportance) structured pruning strategy, significantly improving performance in scenarios with tiny disease targets and limited edge computing resources [20].

Existing lightweight small-object optimization strategies for detecting tiny agricultural diseases and pests can be broadly categorized into three types. Among them, inference- or training-level image slicing strategies, such as SAHI, do not require modifications to the network architecture and are generally regarded as post-processing or data augmentation approaches. SAHI divides high-resolution original images into overlapping small patches, thereby enlarging tiny targets and increasing their pixel-level proportion. Since it does not modify the backbone or detection head, it has a very low deployment cost and has been widely applied to UAV-based pest detection and high-altitude remote-sensing small-object detection tasks [21,22]. However, SAHI has several inherent limitations. First, sliced inference greatly increases image inference time; after high-resolution greenhouse RGB images are divided into patches, the processing latency of a single image increases substantially, making it difficult to satisfy the 45 FPS real-time monitoring requirement for greenhouse deployment. Second, image slicing may truncate overlapping diseased regions between leaves or small lesions located near patch boundaries, resulting in missed detections of cropped targets. Third, SAHI only improves the image-level pixel scale and cannot enhance weak lesion textures or extremely low-contrast targets at the network feature level; therefore, its ability to improve robustness against strong greenhouse illumination, leaf occlusion, and complex soil backgrounds remains limited. Another category is lightweight single-module receptive-field expansion methods, represented by YOLOFs and LRFL-YOLO, which enlarge the receptive field through individual modules such as dilated convolution or separable large-kernel convolution, while introducing only a small number of additional parameters and requiring relatively simple structural modifications [23]. Nevertheless, such methods mainly address the lack of global contextual information and do not specifically resolve the three core challenges in tiny agricultural object detection: the loss of shallow small-lesion information caused by downsampling, insufficient cross-layer feature reuse, and interference from complex greenhouse background noise. When used alone, these methods provide only limited improvement for early-stage strawberry lesions smaller than 16 pixels, with mAP typically increasing by only 3–6%, which remains insufficient to meet the accuracy requirements of early disease-warning systems in greenhouse environments.

Nevertheless, despite the remarkable achievements of various YOLO series models and their improved variants in crop disease and pest detection, strawberry disease and pest detection in greenhouse visual sensing scenarios still faces several critical challenges. First, early-stage strawberry lesions are extremely small and contain limited texture information, making them vulnerable to information loss and missed detection during deep downsampling. Second, dense leaves, branch occlusions, and soil background interference severely affect the robustness of detection models in sensor-captured greenhouse images. Third, the high proportion of tiny lesions and the uneven distribution of target scales pose significant challenges for single-scale or shallow-feature-based detection networks [24,25,26].

To address these issues, this study proposes YOLOv8n-DSLW, a deployment-oriented AI-enabled visual detection model for tiny strawberry disease and pest detection in greenhouse images. The main contributions are summarized as follows:

(1) Dense Blocks (ShrinkRDB) are introduced into the backbone network, while the C2fSA module is integrated into the FPN and PAN structures to achieve cross-layer feature reuse and channel–spatial attention filtering. From the perspective of greenhouse visual sensing, this design enhances the preservation of weak tiny-lesion textures in sensor-captured images while suppressing interference from complex canopy backgrounds, leaf occlusion, and uneven illumination [27].
(2) A high-resolution P2 detection layer is added to strengthen tiny-target perception, and the WIoU dynamic regression loss function is introduced to optimize bounding box localization through dynamic focusing and outlier-degree evaluation. This strategy improves the integrity and localization accuracy of early-stage tiny lesions and small pest targets that are easily weakened during repeated downsampling in camera-based greenhouse monitoring scenarios [28].
(3) The LSKA module is embedded after the SPPF layer to enhance global contextual perception and expand the effective receptive field of the model. This design improves the responsiveness of the visual detection model to tiny strawberry disease and pest symptoms under complex greenhouse backgrounds, dense foliage, and scale variations, thereby strengthening global–local feature representation for AI-enabled crop health sensing [29].
(4) The Layer-Adaptive Magnitude-based Pruning (LAMP) strategy is adopted to reduce redundant model parameters and achieve model lightweighting while maintaining detection accuracy. This pruning strategy improves the accuracy–efficiency balance of the proposed model, making it more suitable for deployment-oriented greenhouse visual monitoring and resource-constrained agricultural sensing devices.

## 2. Materials and Methods

The core workflow of strawberry disease and pest visual detection in this study consists of dataset collection and screening, image preprocessing, algorithm optimization, experimental test, and performance evaluation [30]. A self-collected strawberry disease and pest image dataset acquired from greenhouse visual sensing scenarios in 2025 was established in this study. All images were annotated in COCO format, and images containing different disease categories were screened to construct the initial dataset. Subsequently, image preprocessing operations, including color transformation, Gaussian noise injection, and image flipping, were performed to establish a standardized dataset suitable for AI-enabled visual detection model training [31]. On this basis, key hyperparameters were determined through preliminary experiments, and the detection algorithm was further optimized. Ablation experiments and comparative experiments were conducted to verify the rationality of the proposed model design and demonstrate its detection performance. Finally, various evaluation metrics were analyzed to test the detection effectiveness and practical applicability of the proposed model under complex greenhouse visual sensing conditions.

### 2.1. Dataset Construction

#### 2.1.1. Greenhouse Strawberry Disease and Pest Image Dataset

The experimental dataset consisted of field-collected strawberry plant images from greenhouse environments. All image samples in this dataset were manually collected by the research team from multiple solar greenhouses in Xi’an, Shaanxi Province, during the whole growing season of strawberries in 2025. The collection period spanned from early March to late July 2025, covering the flowering, fruit setting, and mature fruiting stages of strawberries, which comprehensively gathered disease and pest lesion samples generated under natural high-temperature and high-humidity greenhouse cultivation conditions. The images were acquired using a Canon EOS 80D (Canon Inc. Production location: Ōita, Japan) consumer-grade red–green–blue (RGB) digital camera, and the image resolution was 1266 × 873 pixels. The dataset included 3000 images categorized into seven classes: Angular Leaf Spot, Anthracnose Fruit Rot, Blossom Blight, Gray Mold, Leaf Spot, Powdery Mildew (Fruit), and Powdery Mildew (Leaf). The images were collected from strawberry plants at different growth stages and contained multi-scale targets, complex backgrounds, and diverse illumination conditions, thereby effectively reflecting practical greenhouse image acquisition environments. The Common Objects in Context (COCO) JavaScript Object Notation (JSON) annotation format was employed for model training. Each image contained one or more disease regions annotated using axis-aligned bounding boxes. The dataset used in this study, including the training and independent test subsets, has been uploaded to a GitHub repository for dataset verification and is available at: https://github.com/dataset-review-2026/strawberry-dataset (accessed on 26 July 2026).

To ensure sample independence and avoid data leakage, all images in the dataset were collected following the one image per plant principle: each image corresponds to an independent strawberry plant, and no plant was sampled repeatedly from multiple angles or periods. All dataset partitions are based on plant-level units, ensuring completely mutually exclusive samples among the training, validation, and test sets.

A full dataset duplicate check was performed using the perceptual hash (pHash) algorithm with a Hamming distance threshold of 5. The results confirm that there are no completely identical or highly similar duplicate images in the dataset. The detailed statistics are shown in Table 1.

It should be noted that all images in this dataset were captured manually at close range using professional cameras under relatively controlled shooting conditions. There are discrepancies in imaging qSSuality, shooting angles, environmental interferences and other aspects between such images and those captured by fixed monitoring devices actually deployed in greenhouses. The detection performance obtained based on this dataset can reflect the theoretical upper limit of the algorithm, and its performance in real deployment scenarios needs to be further verified with actual hardware.

#### 2.1.2. Image Preprocessing and Data Augmentation

This study was based on 3000 original strawberry disease and pest images collected from field greenhouse environments. Standardized preprocessing and data augmentation were conducted in 2025 to improve the diversity of the dataset. When disease images are acquired using agricultural detection devices, visual detection under real greenhouse conditions is highly susceptible to illumination variations, restricted shooting angles, and interference from high-temperature and high-humidity microclimatic environments. Therefore, data augmentation was introduced in this study to improve the adaptability of the model to sensor-acquired greenhouse images, alleviate sample category imbalance, and enhance its generalization ability under complex environmental conditions [32].

The 3000 strawberry disease and pest images were first randomly divided into a training set and an independent test set at a ratio of 9:1, resulting in 2700 training images and 300 test set images. On this basis, 10% of the raw training images (270 images) were further randomly extracted as an independent validation set, and the remaining 2430 images were used as the formal training set. All offline data augmentation was applied only to the formal training set images. The validation set and test set retained their original images without any augmentation processing, and no data exchange or sample overlap occurred among the three sets. This hierarchical partitioning strategy strictly ensures that neither the validation set nor the test set participates in model weight update and data augmentation, fundamentally eliminating the risk of data leakage. After offline data augmentation, the total number of images increased from 3000 to 55,326. The dataset contained 5462 annotated disease bounding boxes in total, among which 3817 were tiny lesions with an area smaller than 1024 px^2^, accounting for 69.9%; 1425 were medium-sized lesions, accounting for 26.1%; and only 220 were mature large lesions, accounting for 4.0%. A large number of early-stage powdery mildew and anthracnose spots were only 8 × 8 to 16 × 16 pixels in size, representing typical extremely small targets. Even though the images were captured at close range, dense leaf stacking and multi-layer occlusion still substantially compressed the effective pixels of the lesions, resulting in detection difficulties comparable to those encountered in aerial or long-distance small-object detection. After augmentation, the dataset consisted of 5026 training images and 300 test images. According to leaf occlusion, background interference, and illumination fluctuation, all images were further divided into three scene categories: simple scenes, accounting for 22.3%, characterized by single leaves, no occlusion, uniform illumination, and only a small number of mature large lesions; moderate scenes, accounting for 47.5%, characterized by slight overlap among multiple leaves, interference from soil and withered branches, and a mixture of lesions of different sizes; and complex high-difficulty scenes, accounting for 30.2%, characterized by multi-layer leaf occlusion of lesions, backlighting or shadows, strong overexposure, and numerous fine withered-leaf and soil-like background spots, where tiny lesions exhibit highly similar grayscale characteristics to the background and are therefore prone to missed detections and false detections.

The original greenhouse strawberry images collected using a Canon EOS 80D digital camera had a resolution of 1266 × 873 pixels. The image width and height were unequal, and their aspect ratio differed from the model input specification. To standardize the image input dimensions during network training and inference and to eliminate scale interference caused by inconsistent image sizes, a standardized resolution scaling procedure was designed in the preprocessing stage as follows:

(1) Proportional resizing and edge padding

All original images with a resolution of 1266 × 873 were first resized while preserving their original aspect ratio. The longer side of each image was scaled to 640 pixels, while the shorter side was padded with gray blank pixels, ultimately converting all images into a unified 640 × 640 square format. This processing strategy avoids texture distortion of tiny lesions caused by direct stretching and fully preserves the spatial information of early-stage small lesions and occluded pest targets.

(2) Synchronous mapping of annotation box coordinates

During the resizing and padding transformation, the pixel coordinates of the COCO-format bounding-box annotations were recalculated according to the image scaling ratio. The position and size of each bounding box after resizing were automatically updated, ensuring that annotation misalignment and target displacement would not occur after resolution conversion.

(3) Unified model input specification

After resizing and padding, both the training and test images were fixed at 640 × 640 pixels as the network input resolution, consistent with the hyperparameter settings listed in Table 2. The unified input size stabilizes the feature extraction stride of the backbone network, ensures the stability of the P2/P3/P4/P5 multi-scale pyramid feature fusion process, and reduces training oscillations caused by fluctuations in image resolution.

**Figure 1:**
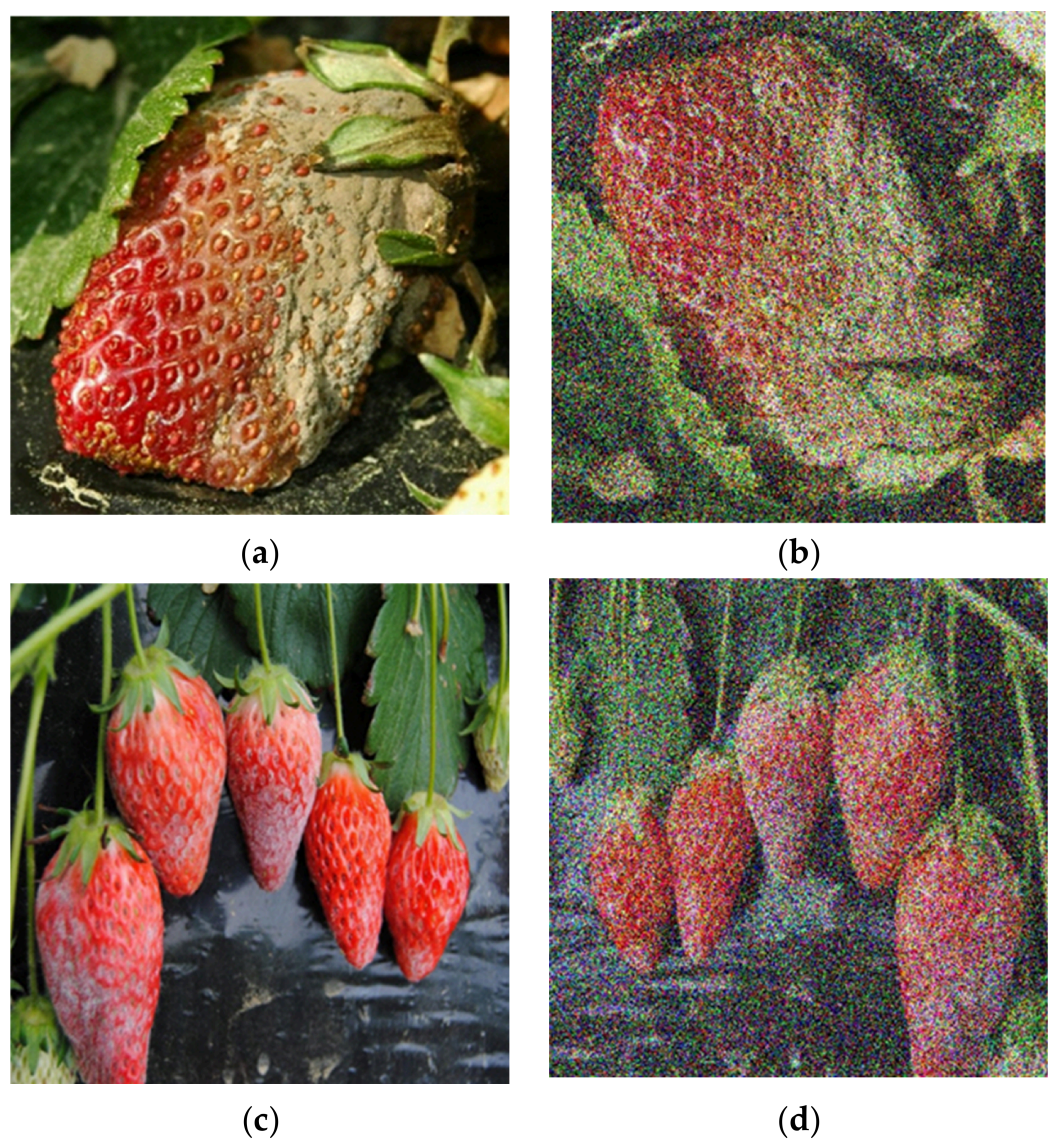
Visualization of Offline Data Augmentation Results: (**a**) Before Offline Data Augmentation; (**b**) After Offline Data Augmentation; (**c**) Before Offline Data Augmentation; (**d**) After Offline Data Augmentation.

**Figure 2:**
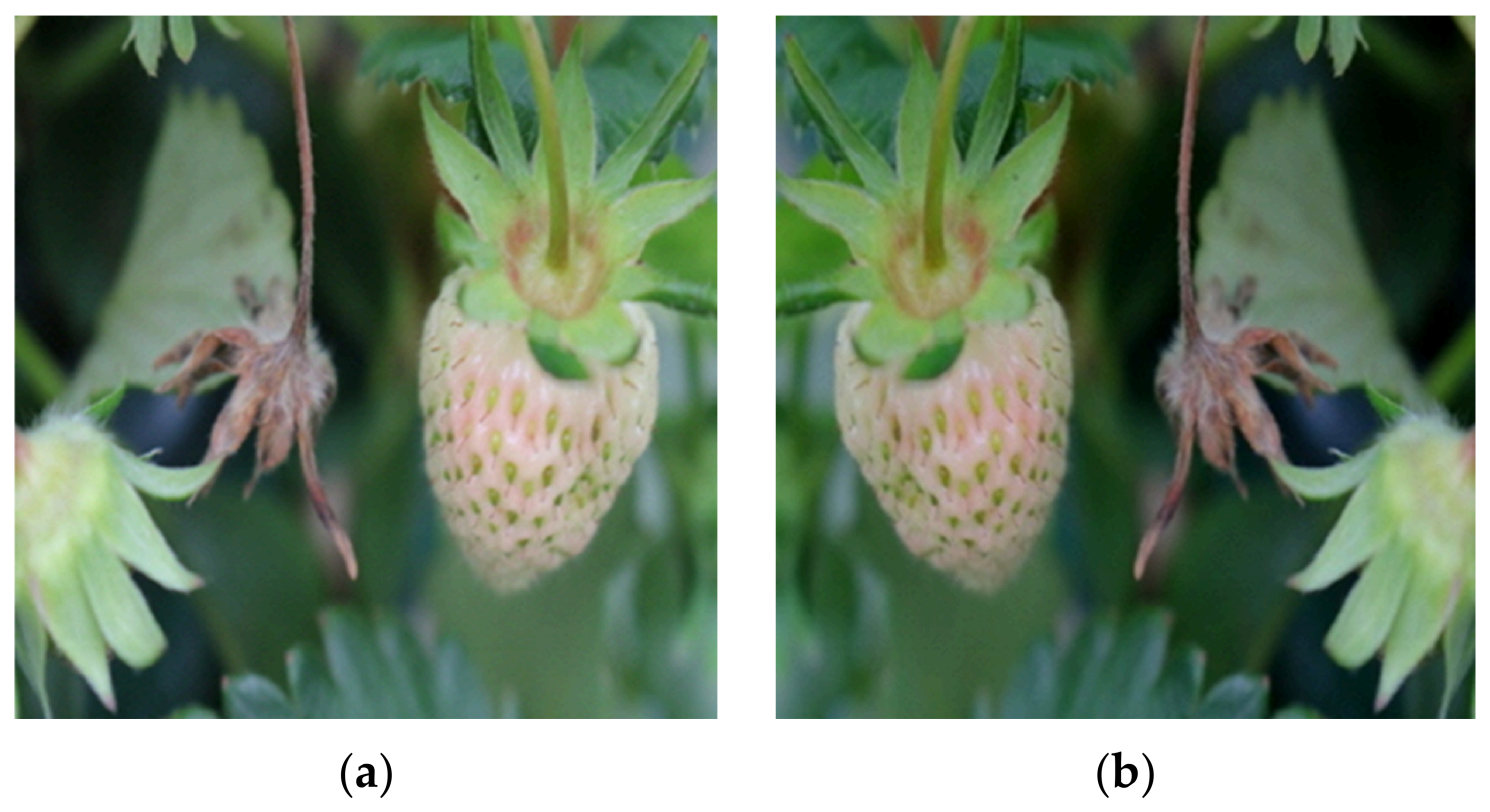
Spatial Transformation Illustration: (**a**) Before Spatial Transformation; (**b**) After Spatial Transformation.

**Figure 3:**
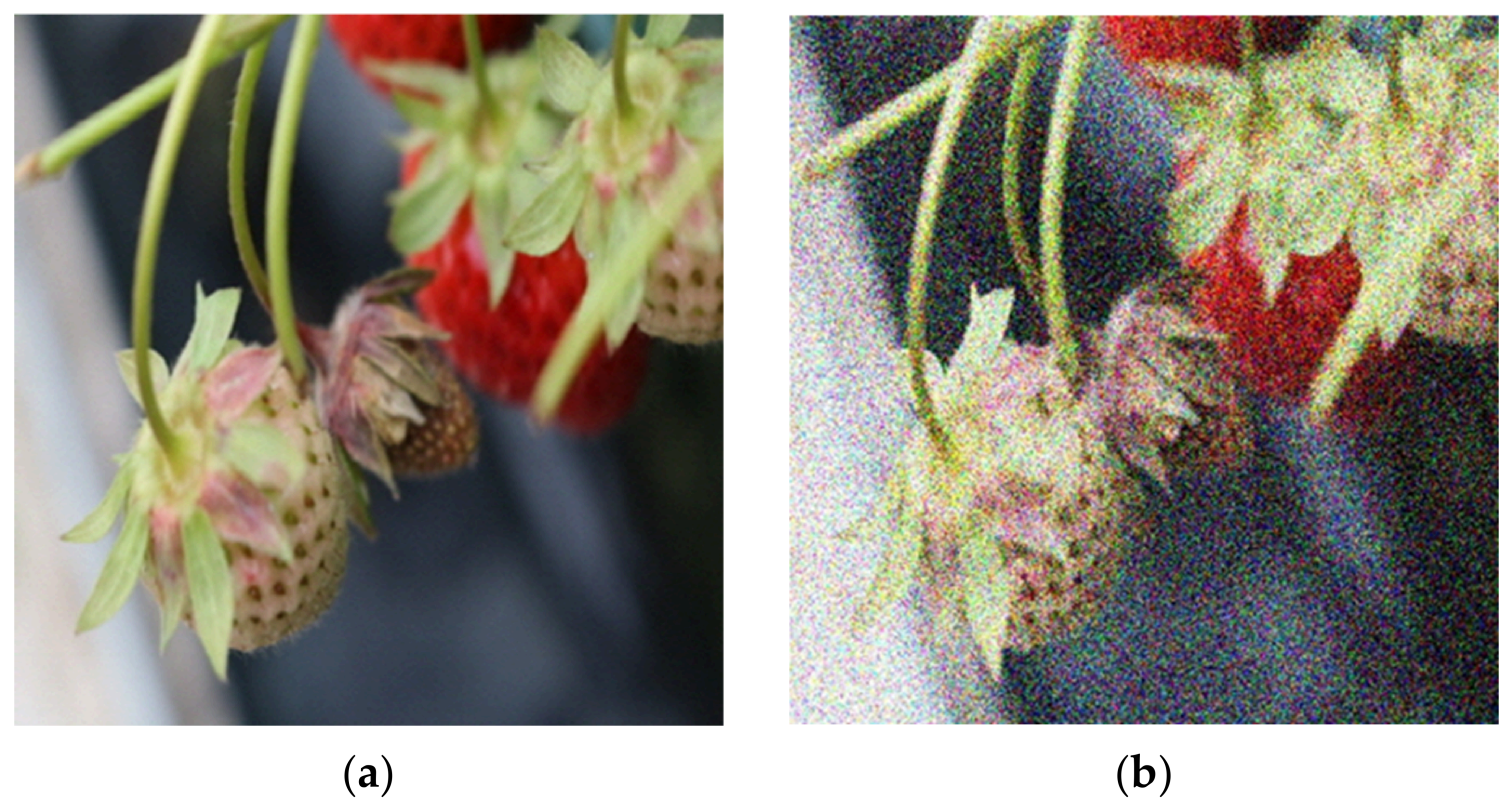
Gaussian Noise Injection Illustration: (**a**) Before Gaussian Noise Injection; (**b**) After Gaussian Noise Injection.

**Figure 4:**
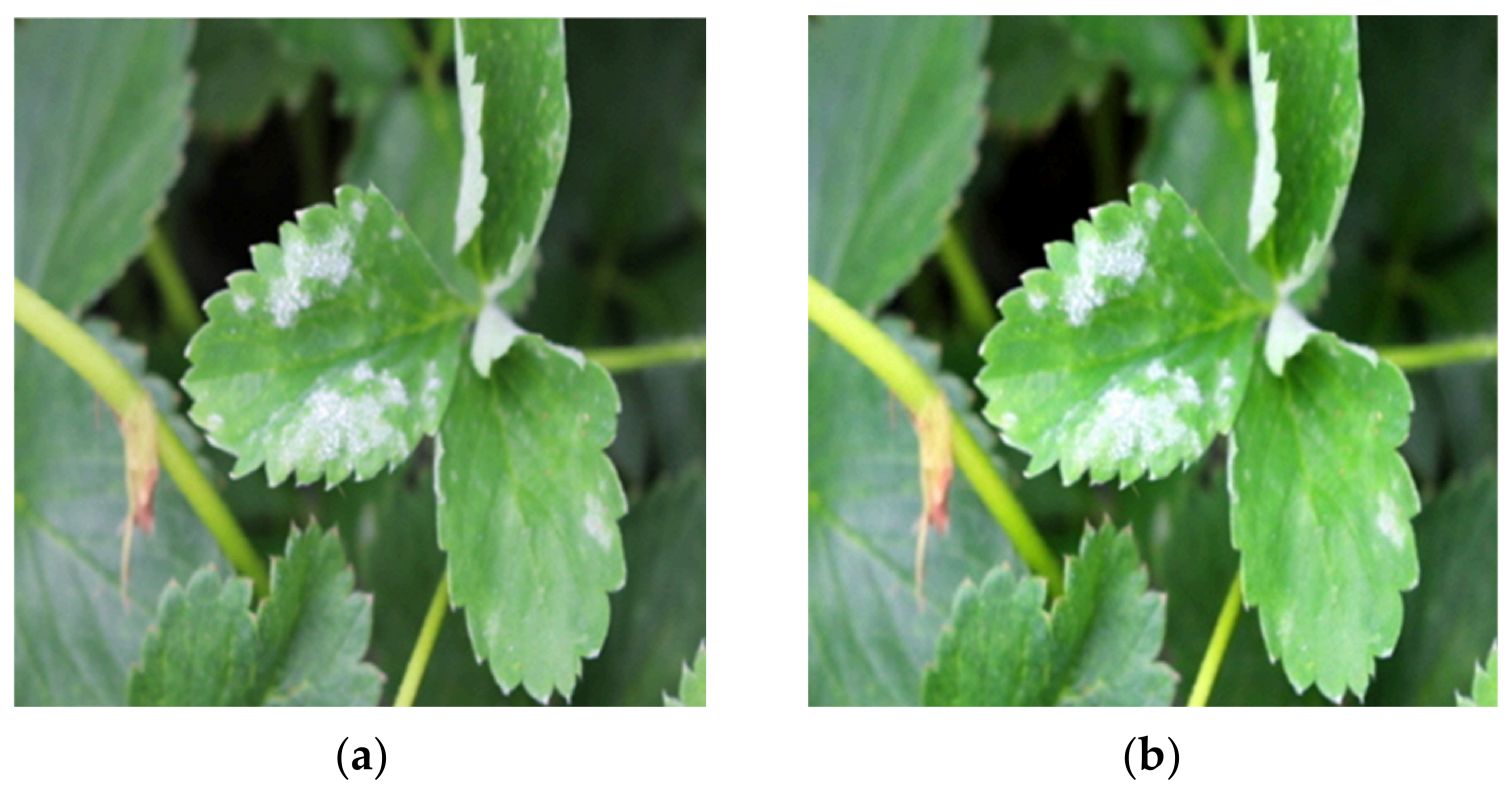
Color Transformation Illustration: (**a**) Before Color Transformation; (**b**) After Color Transformation.

Offline data augmentation techniques were adopted in this study. Specifically, 50% of the training images were subjected to color transformations, including random adjustments of brightness, contrast, and saturation, as well as Gaussian noise injection and spatial transformations. These operations were used to simulate common variations in sensor-captured greenhouse images, such as illumination fluctuation, color shift, mild image noise, and viewpoint changes. The spatial transformations included horizontal flipping with a probability of 50% and vertical flipping with a probability of 50%. When bidirectional spatial flipping was randomly triggered, the bounding box coordinate synchronization mechanism was simultaneously applied to ensure annotation consistency. The preprocessing results are illustrated in Figure 1, Figure 2, Figure 3 and Figure 4.

In addition, online data augmentation strategies were employed during model training, including Mosaic augmentation and hue–saturation–value (HSV) color fine-tuning. Mosaic augmentation enhanced the model’s capability to capture extremely tiny lesions under complex greenhouse backgrounds, while HSV color fine-tuning improved the robustness of the model to color and illumination variations in sensor-captured images.

### 2.2. YOLOv8n-DSLW

To address the challenges of strawberry disease and pest detection in sensor-captured greenhouse images, including numerous tiny lesions, complex backgrounds, illumination variations, and dense canopies, a lightweight and high-precision AI-enabled detection model, termed YOLOv8n-DSLW, was constructed based on the YOLOv8n framework. The proposed model optimizes the network architecture from four aspects: lightweight feature extraction, multi-scale tiny-target enhancement, attention-based background suppression, and deployment-oriented model pruning. While maintaining practical inference efficiency, the proposed model aims to improve the detection accuracy of tiny lesions and the robustness of visual perception under complex greenhouse conditions.

#### 2.2.1. Lightweight Feature Extraction Network Integrating Dense Connections and Shuffle Attention

**Figure 5:**
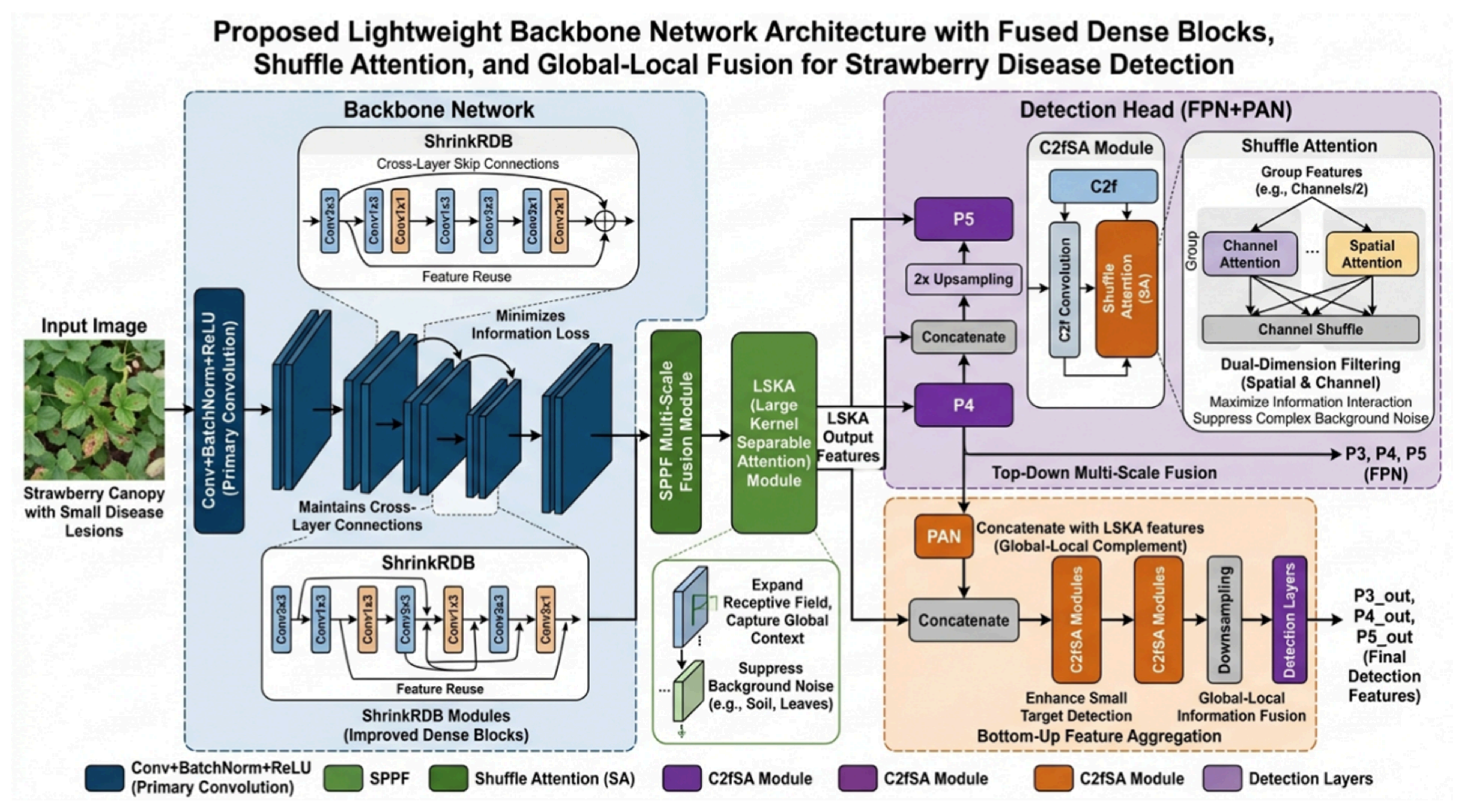
Overall architecture of the proposed YOLOv8n-DSLW model, including backbone with ShrinkRDB dense blocks, SPPF-LSKA multi-scale fusion module, and C2SA-enhanced FPN-PAN detection neck.

To achieve efficient feature extraction and detail preservation for tiny disease and pest targets within dense strawberry canopies, a lightweight backbone network integrating Dense Blocks and Shuffle Attention was proposed in this study. Figure 5 illustrates the overall network architecture of YOLOv8n-DSLW. From top to bottom, the figure presents three main components: (1) a lightweight backbone feature extraction network embedded with ShrinkRDB dense residual blocks; (2) an SPPF-LSKA global contextual enhancement module; and (3) an FPN+PAN multi-scale detection neck integrated with Shuffle Attention (C2fSA). The backbone network represents only the front-end submodule of the overall architecture.

After the input image passes through the initial convolution layers of the backbone network to extract low-level visual features, the feature maps are fed into the ShrinkRDB module, which is improved based on Dense Blocks. Through multi-layer convolution and cross-layer direct connections, the module achieves efficient feature reuse and maximally preserves the detailed texture information of early-stage strawberry lesions. During the middle and deep feature extraction stages, each Conv+ShrinkRDB unit continuously maintains cross-layer connectivity, thereby enhancing feature information flow and reducing effective information loss.

After multi-scale fusion through the SPPF module, the extracted features are further processed by the Large Separable Kernel Attention (LSKA) module. By enlarging the receptive field, the LSKA module captures global contextual information and suppresses background noise interference caused by soil, overlapping branches, and leaves in field environments [33].

For the detection head, the C2fSA module is integrated into both the Feature Pyramid Network (FPN) and the Path Aggregation Network (PAN) structures.

In the FPN branch, a top-down multi-scale feature fusion strategy is adopted. The P5 feature maps generated by the backbone network are upsampled by a factor of two and concatenated with the P4 feature maps. The fused features are subsequently processed by the C2f convolution block to extract composite features. Afterwards, Shuffle Attention performs channel shuffling operations to reconstruct channel features, enabling cross-group information interaction while reducing computational overhead.

In the PAN branch, a bottom-up feature aggregation strategy is employed. The downsampled high-resolution features are sequentially processed by the C2f convolution block and Shuffle Attention module. Combined with the output features from the LSKA module, global and local information complement each other, thereby improving the detection performance for tiny disease targets.

Traditional Convolutional Block Attention Module (CBAM) mechanisms generally rely on serial extraction of channel and spatial attention. However, this linear stacking strategy introduces substantial computational overhead. To overcome this computational bottleneck, the Shuffle Attention mechanism is introduced in this study for grouped parallel processing [34]. The module simultaneously filters effective features in both spatial and channel dimensions, making it more suitable for capturing fine-grained local details required in strawberry tiny disease detection tasks.

In summary, the structural integration of Dense modules and the Shuffle Attention mechanism enables complementary advantages. The Dense module maximizes cross-layer feature utilization and preserves detailed textures of early-stage lesions, while Shuffle Attention optimizes feature maps in both spatial and channel dimensions, alleviating the redundant features and noise introduced by dense connections. Consequently, the proposed model achieves a balance between feature representation capability and computational efficiency, enabling lightweight architecture design and real-time inference while accurately distinguishing visually similar disease categories.

#### 2.2.2. Collaborative Detection Mechanism Based on P2 Feature Enhancement and WIoU Dynamic Regression

As a critical component connecting the feature fusion network and the final prediction output, the detection head directly affects the model’s capability to discriminate target categories. The YOLO series models adopt a hierarchical output structure to accommodate multi-scale object detection tasks. In the default configuration of YOLOv8, three detection layers, namely P3, P4, and P5, are employed for small-, medium-, and large-scale target extraction and localization, respectively. However, when detecting tiny targets such as early-stage strawberry lesions and subtle pest traces, shallow feature details are prone to being lost during repeated downsampling operations, leading to degraded detection performance for small objects. Specifically, the original P3 detection layer in YOLOv8 adopts a downsampling stride of 8, which easily causes irreversible smoothing loss of geometric details for tiny targets, such as early strawberry powdery mildew spots, before entering the detection head [35].

To address the above issue, a P2 feature enhancement branch was constructed in this study. After the backbone network performs convolution operations with a stride of 4, the output features are directly extracted to generate high-resolution P2 feature maps, which retain abundant edge and texture information. Subsequently, the P2 features are fed into the neck network and concatenated with the upsampled deep features generated by the backbone network for feature fusion. Meanwhile, the channel number of the P5 large-target detection layer is reduced, allowing computational resources to be reallocated toward shallow features. This strategy guides the model to focus more on small and tiny targets, thereby improving the perception accuracy of target existence [36].

**Figure 6:**
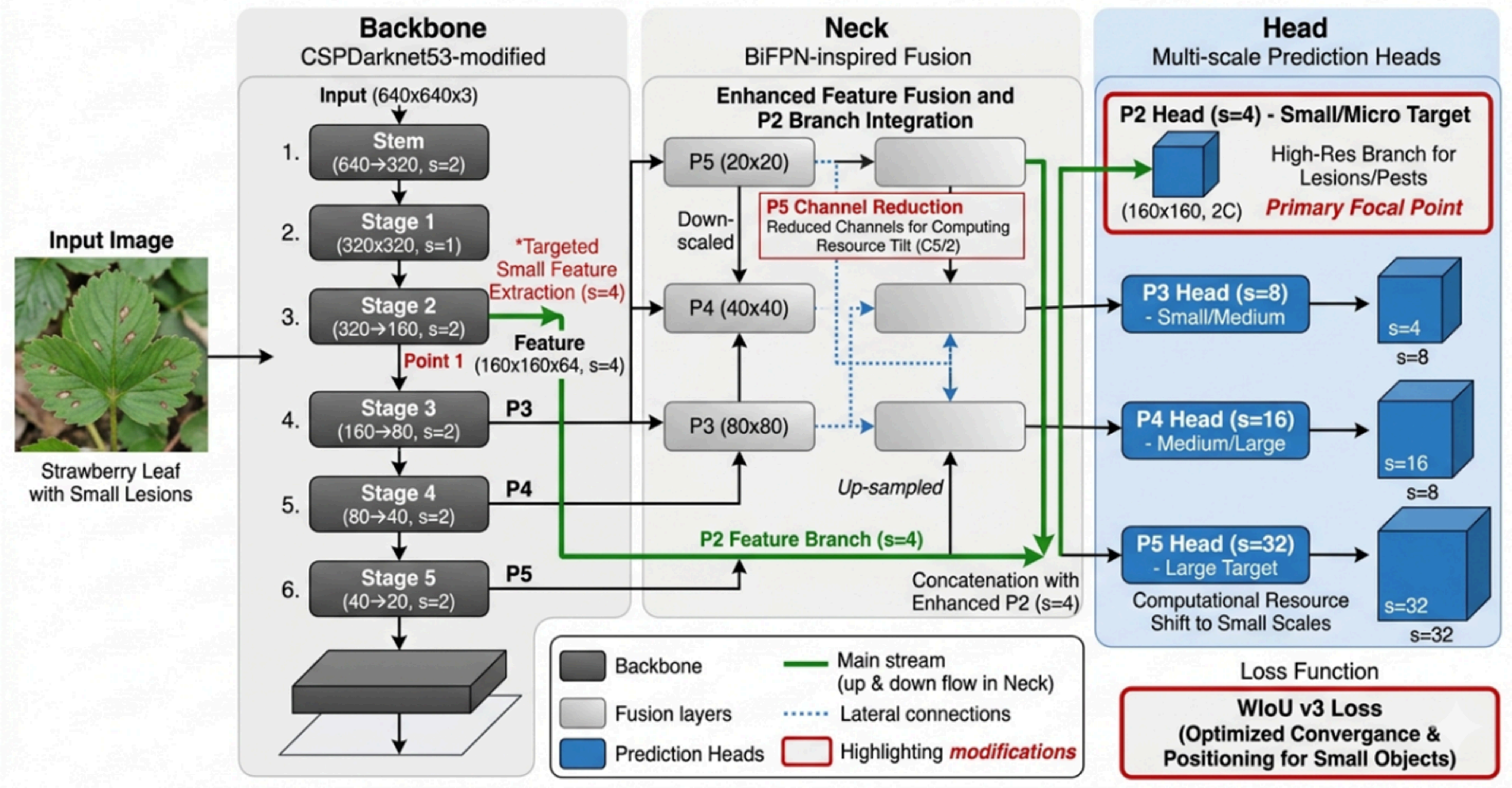
Modified YOLOv8 with P2 Enhancement and WIoU Loss for Micro Strawberry Pest and Disease Detection.

Although the P2 layer can effectively highlight tiny target features, complex greenhouse illumination conditions and leaf occlusions may simultaneously introduce substantial background noise. In addition, the traditional Complete Intersection over Union (CIoU) loss function is highly sensitive to low-quality samples, which easily leads to bounding box localization deviation. To overcome these limitations, the WIoU v3 loss function was introduced in this study to optimize the convergence efficiency and localization accuracy of the model [37]. The corresponding network architecture is illustrated in Figure 6.

The WIoU loss adopts a dynamic adaptive non-monotonic focusing mechanism. During the regression process of prediction boxes generated by the P2 layer, the quality of anchor boxes is first dynamically evaluated, and the outlier degree parameter *β* is subsequently calculated as follows:

(1) $$\beta \;=\;\frac{L_{\mathrm{IoU}}}{L_{\bar{\mathrm{Io}}U}}\in \left[0,+\infty \right)$$

Among them, $L_{\mathrm{IoU}}$ represents the IoU loss of a single prediction box, while $L_{\overset{¯}{\mathrm{Io}}U}$ denotes the sliding average of the IoU loss.

After the outlier degree evaluation is completed, spatial deviations still exist between the predicted bounding box and the ground-truth bounding box. Therefore, the Wise-IoU penalty term is further introduced as follows:

(2) $$R_{WIoU}=exp\left(\frac{{\left(x-x_{gt}{)}^{2}+(y-y_{gt}\right)}^{2}}{{\left(W_{g}^{2}+H_{g}^{2}\right)}^{*}}\right)$$

Among them, (x,y) and $\left(x_{gt},y_{gt}\right)$ represent the center coordinates of the predicted bounding box and the ground-truth bounding box, respectively. $W_{g}$ and $H_{g}$ denote the width and height of the minimum enclosing rectangle covering both the anchor box and the ground-truth box. The superscript ∗ indicates that the corresponding denominator term does not participate in backpropagation, which effectively suppresses gradients that may lead to training instability.

Finally, the WIoU loss is defined as follows:

(3) $$L_{\mathrm{WIoU}}=r\cdot R_{\mathrm{WIoU}}\cdot L_{\mathrm{IoU}}$$

Among them, *r* denotes the focusing parameter, which automatically adjusts the weighting coefficient according to the value of *β*. The corresponding expression is defined as follows:

(4) $$r=\frac{\beta }{\delta \alpha^{\beta -\delta }}$$

In Equation (4), δ and α are manually predefined hyperparameters. δ controls the threshold for judging outlier samples, and α adjusts the variation rate of focusing parameter r with the outlier degree β. In all our experiments, we set δ = 1.0 and α = 1.9. When the outlier degree β of the prediction box is larger than δ, the model reduces the loss weight of such low-quality samples to mitigate the training interference caused by offset targets from occluded and tiny lesions. When (β < δ), the model increases the weight of medium-quality anchor boxes to strengthen localization convergence for tiny disease lesions.

In summary, the P2 feature enhancement branch and the WIoU dynamic regression strategy jointly form a collaborative detection mechanism. The P2 layer preserves the spatial details of tiny targets, thereby alleviating the missed detection problem. Meanwhile, the WIoU loss addresses localization deviation and background noise interference in complex scenarios through dynamic gradient allocation and outlier degree filtering. This mechanism enables the model to focus more effectively on medium-quality anchor boxes, significantly improving localization accuracy and convergence stability, and thereby providing reliable technical support for high-density strawberry disease and pest detection.

#### 2.2.3. Multi-Scale Feature Fusion and Background Suppression Based on the Collaboration of SPPF and LSKA

Considering the complex background of strawberry leaves and the irregular morphology of disease regions, the LSKA mechanism is introduced in this study. While maintaining the capability of capturing effective receptive fields, the proposed module significantly reduces parameter quantity and computational overhead through a lightweight design. Consequently, the model’s perception ability for tiny lesion features is effectively enhanced, making it more suitable for real-time agricultural vision detection and edge deployment scenarios.

**Figure 7:**
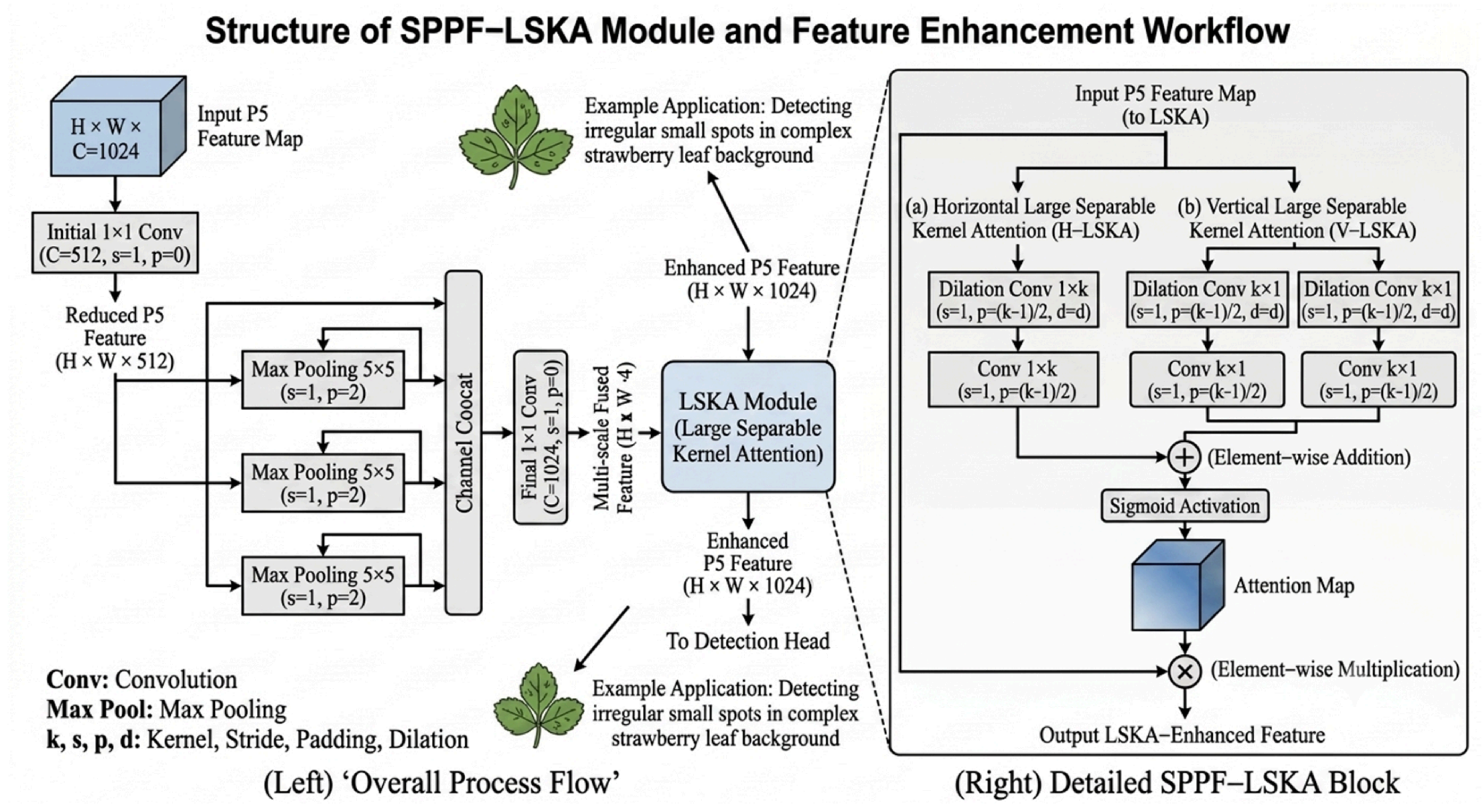
Structure of SPPF-LSKA Module and Feature Enhancement Workflow.

To address the problem that irregular tiny lesion features are easily submerged by complex backgrounds and that multi-scale contextual information is insufficiently utilized, an SPPF-LSKA feature enhancement module was designed in this study [38]. The overall architecture of the proposed SPPF-LSKA module is illustrated in Figure 7. The overall workflow and core structure of the proposed module are described as follows.

(1) Initial 1 × 1 Convolutional Dimensionality Reduction and Channel Compression

The input P5 feature map has a dimension of H×W×1024. First, a 1×1 convolution layer with a stride of 1 and padding of 0 is employed for channel dimensionality reduction, compressing the channel number from 1024 to 512. Without changing the spatial resolution, this operation achieves lightweight channel transformation through linear projection, thereby preserving the core semantic information of the features while effectively reducing the computational complexity of subsequent multi-scale pooling and attention modeling. Consequently, efficient feature representations are provided for the following modules.

(2) Multi-Scale Contextual Feature Extraction Based on the SPPF Structure

The dimensionally reduced feature maps are subsequently fed into the Spatial Pyramid Pooling-Fast (SPPF) structure. Specifically, the features sequentially pass through three max-pooling layers with a kernel size of 5×5, a stride of 1, and padding of 2. After each pooling operation, the current output features are concatenated with the previous features along the channel dimension, thereby constructing multi-branch feature pathways and enabling receptive field modeling at different scales within a single feature map.

In this process, shallow pooling layers focus on enhancing local critical features, whereas deeper pooling layers progressively enlarge the receptive field to capture global contextual dependencies. Subsequently, a 1×1 convolution layer is employed to fuse the concatenated multi-scale features and restore the channel dimension to 1024, generating the multi-scale fused P5 feature representation. This design enables efficient extraction and integration of multi-scale spatial information [39].

(3) Global Attention Enhancement and Background Suppression Based on LSKA

The multi-scale fused P5 features are further fed into the Large-Separable Kernel Attention (LSKA) module to enhance target-related features while suppressing background noise interference. The module consists of horizontal and vertical parallel branches, where one-dimensional dilated convolution and separable convolution are adopted to construct large receptive fields and efficiently model long-range spatial dependencies.

Subsequently, the features generated by the two branches are fused at the element level, and a spatial attention weight map is generated through Sigmoid activation. Finally, the attention weight map is multiplied element-wise with the original input feature map, dynamically enhancing the feature responses of lesion target regions while suppressing interference from complex backgrounds [40].

In strawberry disease and pest detection tasks, this mechanism effectively amplifies the feature signals of tiny lesions and significantly improves the detection recall rate. Meanwhile, through global contextual modeling, multi-scale semantic information is preserved, thereby enhancing the adaptability of the model to complex scenarios.

(4) Feature Output and Connection to the Detection Head

The P5 feature maps enhanced by the LSKA module maintain both the original spatial resolution and channel dimensions and are directly fed into the detection head for subsequent object detection tasks. Through the two-stage enhancement strategy of “multi-scale pooling + global attention,” the SPPF-LSKA module enables the output features to simultaneously possess rich multi-scale contextual information and enhanced responses to critical target regions, thereby providing highly discriminative and robust feature representations for the detection head.

Considering the practical challenges in strawberry disease and pest detection, including complex leaf backgrounds, irregular lesion morphology, tiny early-stage lesions, and weak texture characteristics, the proposed SPPF-LSKA module effectively amplifies the feature signals of tiny lesion regions and suppresses background interference through the collaborative integration of multi-scale feature fusion and large-receptive-field attention enhancement. Meanwhile, the module simultaneously preserves local detail information and global contextual semantics, significantly improving lesion detection recall and localization accuracy while enhancing the generalization capability of the model under varying illumination conditions, leaf postures, and lesion distributions. Therefore, the proposed method provides a lightweight and efficient feature enhancement solution for agricultural disease and pest detection tasks.

#### 2.2.4. Layer-Adaptive Magnitude-Based Pruning Method for the YOLOv8n Model

With the introduction of modules such as Dense Blocks, the P2 detection layer, and LSKA, the number of network parameters and overall model complexity inevitably increase, which may significantly affect real-time inference performance and hinder the deployment of edge devices in greenhouse environments [41]. Considering that disease and pest detection in greenhouse scenarios is generally constrained by limited hardware computing capability and storage resources, traditional magnitude pruning methods typically evaluate parameter importance according to the absolute values of weights and uniformly remove parameters with low weight magnitudes. The corresponding expression can be formulated as follows:

(5) $$s_{i}=|w_{i}|$$

where $w_{i}$ represents the convolutional weight parameter, and $s_{i}$ denotes the corresponding importance score. Smaller weight values indicate a higher probability of being pruned.

To address the above issue, a Layer-Adaptive Magnitude-based Pruning (LAMP) algorithm based on hierarchical normalization was introduced into the YOLOv8n model in this study. By dynamically evaluating the importance of parameters across different layers and adaptively allocating pruning sparsity, the proposed method reduces model complexity as much as possible while maintaining the original detection accuracy, thereby satisfying practical deployment requirements [42].

The algorithmic principles of LAMP are described as follows.

(1) Weight Tensor Flattening

The convolutional weight tensor W(l) of the *l*-th layer is first flattened into a one-dimensional vector:

(6) $$w^{\left(l\right)}=[w_{1}^{\left(l\right)},w_{2}^{\left(l\right)},\ldots ,w_{n_{l}}^{\left(l\right)}]$$

(2) Importance Score Calculation

The weights are sorted in ascending order according to their absolute values, and the importance score of each weight is calculated as follows:

(7) $$\mathrm{score}(u;w)=\frac{w_{u}^{2}}{\sum_{v\geq u}\;w_{v}^{2}}$$

where $w_{u}^{2}$ denotes the squared magnitude of the current weight, and $\sum_{v\geq u}\;w_{v}^{2}$ represents the cumulative squared sum of the current and subsequent weights. score(u;w) indicates the importance score of the corresponding weight parameter.

The LAMP method proposed in this study is a structured convolutional-kernel pruning strategy rather than single-weight unstructured pruning. In the pruning process, each complete convolutional kernel is treated as the minimum processing unit. Low-importance convolutional kernels are directly removed, together with their corresponding channels. Consequently, the channel structure of the pruned network is permanently modified. This design does not rely on sparse inference libraries and can be directly adapted to conventional CPUs and edge-device deployment. The complete pruning procedure consists of four steps:

① The baseline model is fully pretrained for 100 epochs until convergence to obtain stable weights;
② The convolutional weight tensors are extracted layer by layer and flattened into one-dimensional vectors, and the importance scores of all convolutional kernels in each layer are calculated according to Equation (7);
③ The pruning sparsity of each layer is assigned based on a layer-adaptive rule, while an upper pruning limit is imposed on shallow feature layers to protect the fine-grained textures of tiny disease lesions;
④ Low-score convolutional kernels are removed, the channel dimensions of the network are reconstructed, and the pruned model is fine-tuned for 30 epochs with a fixed learning rate to compensate for the accuracy loss caused by pruning.

In this experiment, the global target pruning ratio was uniformly set to 20%. To avoid the loss of shallow tiny-object features, layer-wise constraints were further introduced: the maximum pruning sparsity of shallow backbone convolutional layers was limited to no more than 8%, whereas the maximum pruning ratio of deeper redundant feature layers, including the SPPF module and detection head, was allowed to reach 28%. All pruning-related hyperparameters were kept fixed, and the number of fine-tuning epochs after pruning was uniformly set to 30 epochs. These parameters were all recorded in the training hyperparameter table to ensure full experimental reproducibility.

After LAMP-based layer-adaptive pruning, the number of parameters in the complete four-module fused network was reduced from 5.32 M to 4.386 M, corresponding to an overall parameter reduction of 17.6%. Meanwhile, the total GFLOPs decreased by 20.9%. By eliminating a large amount of redundant convolutional computation, pruning reduced memory usage on edge devices. Combined with subsequent INT8 quantization and convolution–BN operator fusion, it substantially reduced the floating-point computational burden on ARM devices, serving as a key support for achieving real-time inference at 42.3 FPS on Raspberry Pi 5. At the same time, the layer-wise constraint mechanism preserved shallow features associated with tiny disease lesions. When LAMP was used alone, the mAP@0.5 remained at 91.1%, indicating no obvious performance degradation.

When the 8% upper pruning limit for shallow layers was removed and a uniform global pruning ratio of 20% was applied, the comparative experiment showed that the model mAP decreased by 2.1%, while the missed detection rates of tiny pests and early-stage powdery mildew lesions increased significantly. When the global pruning ratio was further increased beyond 25%, shallow texture features suffered irreversible loss, making the model unable to meet the requirements of early disease warning. In addition, structured pruning permanently modifies the channel structure of the network. Therefore, when the model is transferred to other crops or disease datasets with substantially different object scales, the original layer-wise pruning ratios may no longer be suitable, requiring sparsity re-adjustment and resulting in higher cross-scenario transfer costs.

LAMP achieves adaptive normalization through the ratio between the squared magnitude of the current weight and the cumulative squared sum of subsequent weights. Parameter importance is evaluated according to the obtained scores. A higher score indicates that the corresponding parameter is more important and should therefore be preferentially retained, whereas parameters with lower scores are preferentially pruned.

When the LAMP algorithm is applied to the strawberry disease and pest detection model, two significant advantages can be achieved:

(1) Through intra-layer normalization, critical shallow convolution kernels can be effectively preserved, thereby preventing missed detection of tiny lesions caused by shallow feature loss and ensuring the detection accuracy of small disease regions.
(2) After pruning, the model concentrates computational resources on distinguishing disease regions from healthy plants and leaves, thereby enhancing robustness under complex background conditions and reducing the false-positive detection rate [43].

## 3. Results

### 3.1. Experimental Environment and Parameter Settings

All experiments in this study were conducted under a unified hardware and software environment. The detailed experimental configurations are as follows (Table 3):

### 3.2. Evaluation Metrics

In this study, standard evaluation metrics commonly adopted in object detection tasks were employed, including Precision, Recall, Accuracy, F1-score, Average Precision (AP), Mean Average Precision (mAP), and mAP@0.5. Specifically, Precision represents the proportion of correctly predicted positive samples among all predicted positive samples; Recall denotes the proportion of correctly detected positive targets among all actual positive targets; Accuracy refers to the overall proportion of correctly classified samples among all evaluation samples; the F1-score is the harmonic mean of Precision and Recall; AP represents the average precision and geometrically corresponds to the area under the Precision–Recall (P–R) curve; mAP is defined as the mean value of AP across all target categories; and mAP@0.5 denotes the mAP value when the Intersection over Union (IoU) threshold between the predicted bounding box and the ground-truth box is set to 0.5. Higher values indicate superior overall detection performance.

The corresponding evaluation formulas are expressed as follows:

(8) $$\mathrm{Recall}=\frac{\mathrm{TP}}{\mathrm{TP}+\mathrm{FN}}$$

(9) $$\mathrm{Precision}=\frac{\mathrm{TP}}{\mathrm{TP}+\mathrm{FP}}$$

(10) $$\mathrm{Accuracy}=\frac{\mathrm{TP}+\mathrm{TN}}{\mathrm{TP}+\mathrm{TN}+\mathrm{FP}+\mathrm{FN}}$$

(11) $$F1=\frac{2\times Precision\times Recall}{Precision+Recall}$$

(12) $$\mathrm{AP}=\int_{0}^{1}\;P(R)\mathrm{dR}$$

(13) $$\mathrm{mAP}=\frac{1}{N}\sum_{i=1}^{N}\;AP_{i}$$

where *N* represents the total number of target categories, and $AP_{i}$ denotes the average precision of the *i*-th category. TP (True Positive) indicates that a disease or pest target is correctly detected; FN (False Negative) indicates that a disease or pest target is missed; FP (False Positive) indicates that the background or healthy leaves are incorrectly identified as disease or pest targets; and TN (True Negative) indicates that the healthy background is correctly recognized as disease-free.

### 3.3. Comparative Experiments

To verify the superiority and scenario adaptability of the proposed YOLOv8n-DSLW model in strawberry tiny disease and pest detection tasks, comparative experiments were conducted with current mainstream lightweight YOLO-series object detection models and several representative improved variants of YOLOv8n. All comparative experiments were performed under the unified experimental environment and hyperparameter settings described in Section 3.1 to ensure fairness and reproducibility.

Before the comparative experiments, we further conducted a control experiment to clarify the impact of the validation set on model performance. In the standard experimental scheme adopted in this paper, the validation set was used for training monitoring and early stopping: when the mAP@0.5 on the validation set did not improve for 10 consecutive epochs, the training was terminated automatically to prevent overfitting. In the control experiment without the validation set, all 2700 raw training images were used for training for 100 fixed epochs without early stopping. Both groups of experiments used exactly the same independent test set for performance evaluation. The comparison results are shown in Table 4.

The results show that the performance difference between the two schemes is less than 1%, indicating that the model has good generalization stability. The scheme with the validation set achieves slightly better performance by suppressing overfitting through early stopping. All performance indicators reported in this paper are based on the standard scheme with the validation set, and all final results are tested on the independent test set that never participates in training and validation.

**Figure 8:**
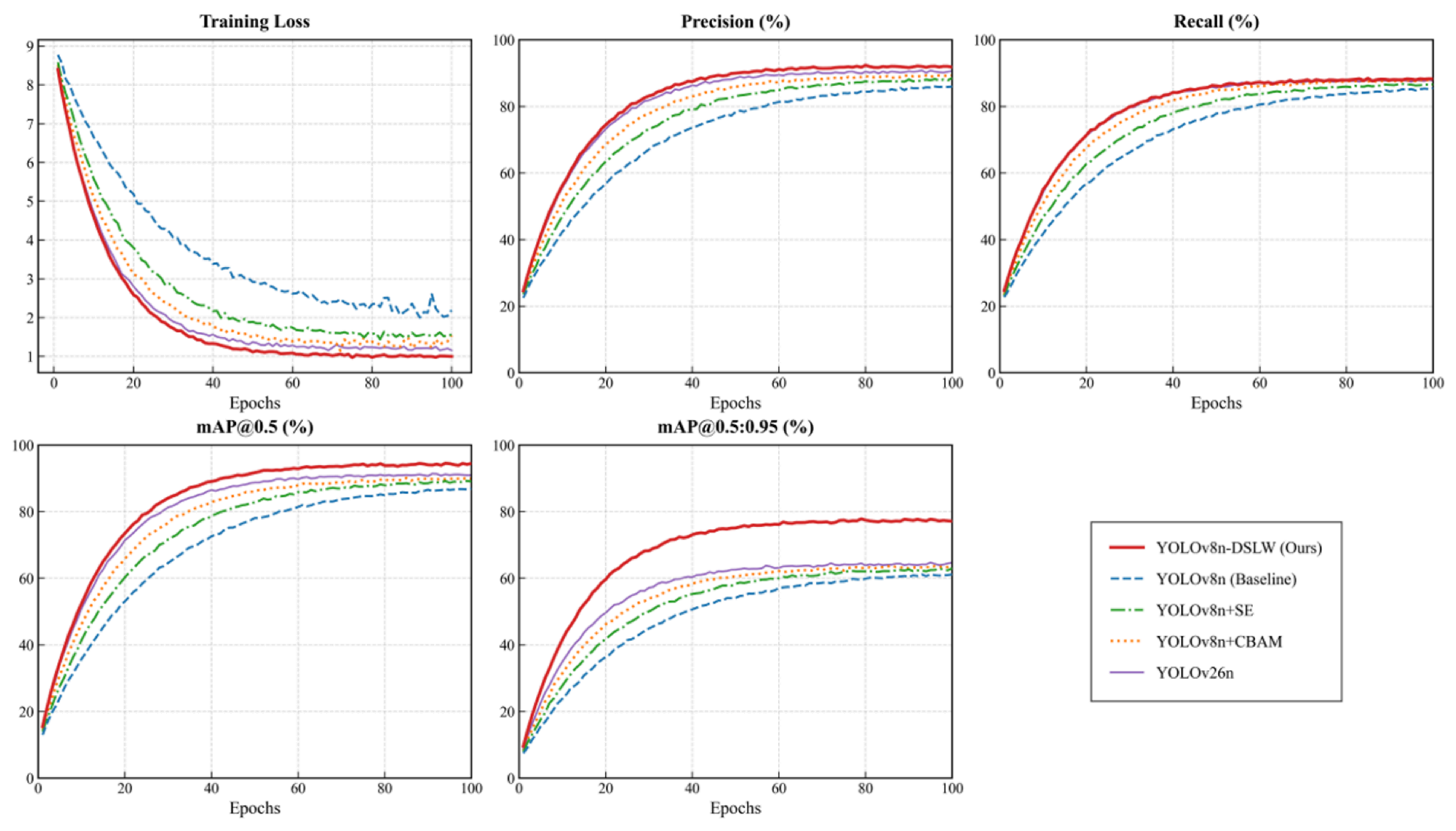
Comparative Results of Different Models.

As illustrated in Figure 8, the training loss curve of the proposed model demonstrates a rapid decline during the early training stage and gradually stabilizes after approximately 70 epochs. Regarding performance metrics, both Precision and Recall continuously improve with increasing training epochs, reaching approximately 92.07% and 88.2% on the test set, respectively. Ultimately, the mAP@0.5 stabilizes at approximately 94.3%, while the mAP@0.5:0.95 stabilizes at approximately 77.5%.

In contrast, the training loss curve of the baseline YOLOv8n model shows a relatively slow decline during the initial training stage, and slight oscillations still persist during the later training stage (after 80 epochs), indicating that the model has not fully reached stable convergence by the end of training. In terms of performance metrics, Precision and Recall only gradually increase with training epochs and eventually reach approximately 87.2% and 81.4% on the test set, respectively. The final mAP@0.5 and mAP@0.5:0.95 stabilize at approximately 91.1% and 62.1%, respectively, which are significantly inferior to those of the YOLOv8n-DSLW model.

For the YOLOv8n+Squeeze-and-Excitation (SE) model, the training loss decreases slightly faster than that of the baseline model during the initial training stage, but it still underperforms the proposed model and gradually stabilizes only after approximately 75 epochs. The Precision and Recall values reach approximately 88.5% and 87.1% on the test set, respectively. The final mAP@0.5 and mAP@0.5:0.95 stabilize at approximately 89.5% and 63.0%, respectively. Although the performance is slightly improved compared with the baseline model, it remains significantly weaker than that of YOLOv8n-DSLW.

The training loss curve of the YOLOv8n+CBAM model demonstrates a faster decline during the early training stage compared with the SE variant and gradually stabilizes after approximately 72 epochs, although slight fluctuations still remain during the later stage. The Precision and Recall values continuously improve with training and eventually reach approximately 89.3% and 88.0% on the test set, respectively. The final mAP@0.5 and mAP@0.5:0.95 stabilize at approximately 90.1% and 63.6%, respectively. Although the performance surpasses both the baseline and SE variants, it still fails to achieve the performance level of the YOLOv8n-DSLW model.

For the YOLOv26n model, the training loss declines rapidly during the initial training stage and gradually stabilizes after approximately 70 epochs, exhibiting a convergence trend similar to that of the proposed model. However, the final loss value remains higher than that of YOLOv8n-DSLW. In terms of performance metrics, Precision and Recall reach approximately 90.5% and 87.9% on the test set, respectively. The final mAP@0.5 and mAP@0.5:0.95 stabilize at approximately 91.2% and 64.3%, respectively. Although YOLOv26n achieves the best performance among the comparative models, its overall performance remains significantly inferior to that of the proposed YOLOv8n-DSLW model.

**Figure 9:**
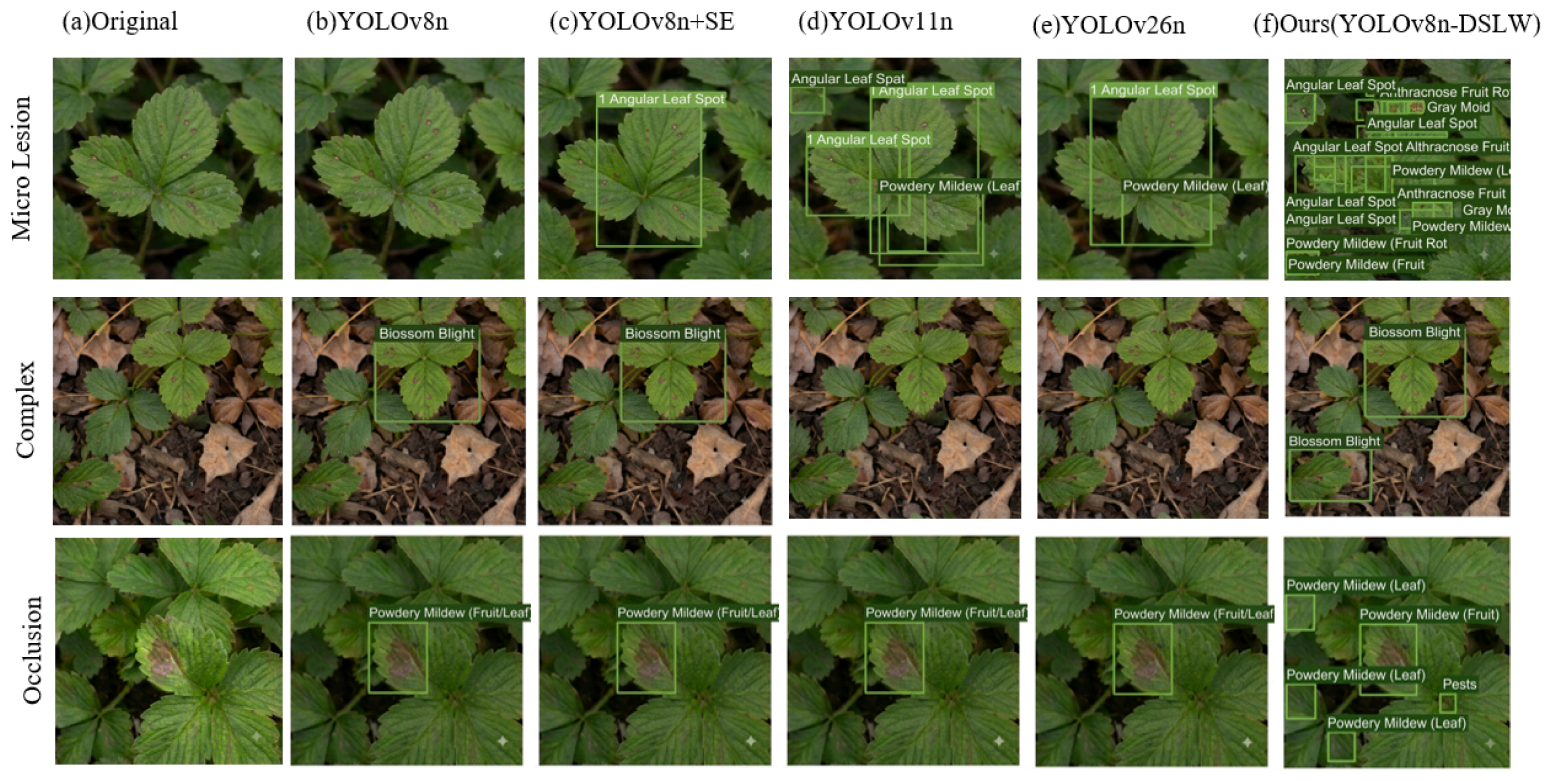
Comparison of Lesion Detection across Models and Scenarios. (**a**) Original image; (**b**) YOLOv8n; (**c**) YOLOv8n+SE; (**d**) YOLOv11n; (**e**) YOLOv26n; (**f**) Proposed YOLOv8n-DSLW.

To visually compare the detection performance of different models, the detection results under three representative field scenarios were selected for qualitative visualization analysis, as illustrated in Figure 9.

Dense Tiny Lesion Scenario: In this scenario, multiple early-stage lesions with sizes smaller than 16 × 16 pixels were distributed on strawberry leaves. Both the original YOLOv8n and YOLOv8n+SE models failed to detect several lesions. Although YOLOv11n and YOLOv26n successfully detected most targets, noticeable bounding box localization deviations still existed. In contrast, the proposed model accurately identified all tiny lesions with higher bounding box fitting accuracy, thereby verifying the effectiveness of the P2 feature enhancement branch and the WIoU loss function for tiny object detection.

Complex Background Interference Scenario: This scenario contained complex background noise, including soil, withered leaves, and shadow interference. Both YOLOv8n and YOLOv8n+CBAM incorrectly identified several background regions as lesion targets. YOLOv11n and YOLOv26n also produced a small number of false-positive detections. Benefiting from the background suppression capability of the Shuffle Attention and LSKA modules, the proposed YOLOv8n-DSLW model effectively distinguished lesion regions from background interference and achieved detection results without false positives.

Leaf Occlusion and Overlapping Scenario: In this scenario, lesion regions were partially occluded by adjacent leaves, resulting in incomplete target features. The original YOLOv8n and YOLOv8n+SE models failed to detect the occluded lesions, whereas YOLOv11n and YOLOv26n detected the targets with relatively low confidence scores. By leveraging the global contextual modeling capability of the SPPF-LSKA multi-scale fusion module, the proposed model successfully captured the critical features of occluded targets and achieved accurate detection with high confidence scores.

To better reflect the hardware conditions of real-world greenhouse monitoring scenarios, this study further conducted edge-device inference experiments on a Raspberry Pi 5 with 8 GB of memory. The hardware platform was equipped with a quad-core ARM Cortex-A76 processor and did not include a discrete GPU. The operating system was Ubuntu 22.04 ARM64, and ONNX Runtime was employed to achieve operator fusion and INT8 quantization acceleration. During the entire testing process, the CPU was fixed in high-performance mode, and background programs were disabled to eliminate interference from computational resource fluctuations. It should be specially clarified that the frame rate data on RTX 3090 and Raspberry Pi are obtained under completely different test configurations and objectives. The RTX 3090 side adopts native FP32 inference without any deployment optimization, which is used to fairly compare the computational complexity of different algorithm architectures. For ultra-lightweight models with only ~4 M parameters, the inference bottleneck on high-performance GPU is CUDA kernel launch overhead and memory access latency, rather than floating-point computing power, so the native frame rate on GPU does not show an order-of-magnitude advantage over the optimized edge-side frame rate. This is a normal technical phenomenon and does not constitute a logical contradiction.

To enable the model to meet the requirements of real-time deployment on low-computing-power devices, a three-level lightweight optimization strategy was adopted: (1) the proposed Layer-Adaptive Magnitude-based Pruning (LAMP) method was used to remove redundant weights through layer-adaptive pruning; (2) INT8 quantization calibration was performed based on the greenhouse disease dataset; and (3) convolution–batch normalization layer fusion was applied to reduce computational cost. All experiments used a unified input size of 640 × 640, and the mAP, frame rate, and peak memory usage were recorded. A frame rate of 40 FPS was used as the threshold for determining whether the model satisfied the real-time monitoring requirement in greenhouse environments.

Table 5 presents a comparison of the inference performance of different models on a desktop GPU and the Raspberry Pi 5 edge device. The native floating-point YOLOv8n-DSLW achieved a very low frame rate on the edge device, making it unsuitable for practical deployment. After the collaborative optimization of LAMP pruning, INT8 quantization, and operator fusion, the model exhibited only a slight decrease in mAP to 93.8%, while achieving a stable inference speed of 42.3 FPS on the Raspberry Pi 5 and reducing memory usage to 376 MB. For the memory reduction magnitude, it should be noted that the theoretical 1/4 volume shrinkage from FP32 to INT8 only applies to model weight parameters. After supplementary verification, the weight file size of the YOLOv8n-DSLW model is reduced from ~21 MB (FP32) to ~4.4 MB (INT8) after quantization, which fully conforms to the theoretical reduction ratio. However, the peak memory counted in this paper covers the full memory overhead of the entire inference process, where model weights only account for about 3%. Most memory is occupied by intermediate feature tensors, inference framework runtime, and system background processes. Since operators such as upsampling and activation still require FP32 precision calculation, and framework runtime and system overhead are not affected by quantization, the overall peak memory reduction is lower than the theoretical value of pure weight quantization, which is consistent with the general law of edge-side model deployment. These results indicate that the optimized model satisfies the deployment requirements for real-time monitoring devices in strawberry greenhouses. Compared with the native YOLOv8n, the optimized DSLW model nearly doubled the edge-device frame rate while improving the recognition accuracy of tiny lesions by 2.7%, thereby achieving a favorable balance between detection accuracy and embedded real-time performance.

To prevent overall aggregated metrics from obscuring differences in the recognition performance of individual disease and pest categories, this study further introduces class-wise AP, Precision, Recall, and a confusion matrix for fine-grained evaluation. The dataset contains seven target categories, including six strawberry disease classes and one tiny pest class consisting of aphids and two-spotted spider mites. In the confusion matrix, the true categories are arranged along the vertical axis and the predicted categories along the horizontal axis, and the numbers of TP, FP, and FN samples for each category are recorded. This representation intuitively reveals category confusion, missed detections, and false detections, enabling targeted analysis of the model’s pest recognition capability and category-specific limitations. The results are presented in Table 6.

### 3.4. Ablation Experiments

To evaluate the contribution of each proposed improvement module to the overall model performance, five groups of ablation experiments were designed in this study. YOLOv8n was employed as the baseline model.

In the first experimental group, the ShrinkRDB module based on Dense Blocks was introduced into the original YOLOv8n model to establish cross-layer direct connections for enhanced feature reuse. Subsequently, Shuffle Attention was applied to the concatenated features within the detection head to achieve cross-group information interaction and improve feature representation capability.

The second experimental group incorporated the P2 small-object detection layer together with the WIoU loss function to enhance the detection accuracy and localization performance of tiny disease targets.

In the third experimental group, the LSKA large-kernel attention mechanism was embedded after the SPPF module to strengthen global contextual perception and suppress background interference under complex field environments.

The fourth experimental group adopted the lightweight Layer-Adaptive Magnitude-Based Pruning (LAMP) strategy to reduce redundant model parameters and computational complexity while maintaining detection performance.

The fifth experimental group integrated all four proposed improvements described above. As a result, the final mAP@0.5 of the proposed YOLOv8n-DSLW model reached 94.3%, demonstrating the effectiveness and complementarity of the proposed optimization strategies. As summarized in Table 7, the results of ablation experiments on different improved modules are presented.

Experiment 1: After introducing the Dense Blocks and Shuffle Attention modules, the mAP@0.5 exhibited a slight decrease, whereas the Recall improved from 0.814 to 0.819. Dense Blocks achieved feature reuse through cross-layer dense connections, thereby reducing repetitive learning of identical information within the network. Meanwhile, Shuffle Attention adopted a lightweight grouped attention mechanism and channel shuffling strategy to effectively suppress background noise interference. Consequently, the number of model parameters was reduced by approximately 23.1%, while the inference speed remained at 80 FPS. These results indicate that the proposed strategy enables the model to capture valid targets more comprehensively while simultaneously balancing lightweight architecture design and high-precision detection performance.

Experiment 2: After convolution operations within the backbone network, the P2 small-object detection layer was introduced for feature enhancement. As a result, the mAP@0.5 increased from 91.1% to 91.3%, while the number of model parameters decreased to 2.927 M, demonstrating notable performance advantages. In addition, replacing the traditional CIoU loss function with the WIoU loss function improved the Recall from 0.814 to 0.851, indicating that the convergence stability of the model during the later training stage was significantly enhanced.

Experiment 3: The LSKA large-kernel attention mechanism achieved significant improvements in detection performance. Specifically, the mAP@0.5 increased to 91.6%, while the F1-score improved from 0.877 to 0.887. These results demonstrate that LSKA effectively enhances the model’s feature perception capability for disease and pest regions and improves target representation performance under complex background conditions. Furthermore, the improved model exhibited superior stability in both the Precision–Confidence and Recall–Confidence curves, thereby verifying the effectiveness of LSKA in enhancing the global information modeling capability of the network.

Experiment 4 introduced only LAMP pruning. In this experimental group, the number of model parameters decreased by 18.46%, while the inference frame rate decreased from the baseline value of 84 FPS to 80 FPS, and the mAP@0.5 remained unchanged at 91.1%. This result can be attributed to the layer-adaptive pruning mechanism. Specifically, the algorithm normalizes the importance scores in a layer-wise manner, restricts the pruning intensity of shallow feature layers, and thereby preserves the fine-grained textures required for detecting tiny disease lesions. As a comparison, an additional supplementary ablation experiment was conducted in this study. A global uniform 20% structured pruning strategy without layer-wise constraints was adopted, while all other conditions remained identical. Under this setting, the model mAP@0.5 decreased to 89.0%, which fully demonstrates the necessity of the layer-wise constraints in LAMP for tiny disease detection. Pure global uniform pruning damages shallow small-object features and fails to balance model lightweighting with detection accuracy. This also serves as the core motivation for designing the layer-adaptive pruning strategy in this study.

**Figure 10:**
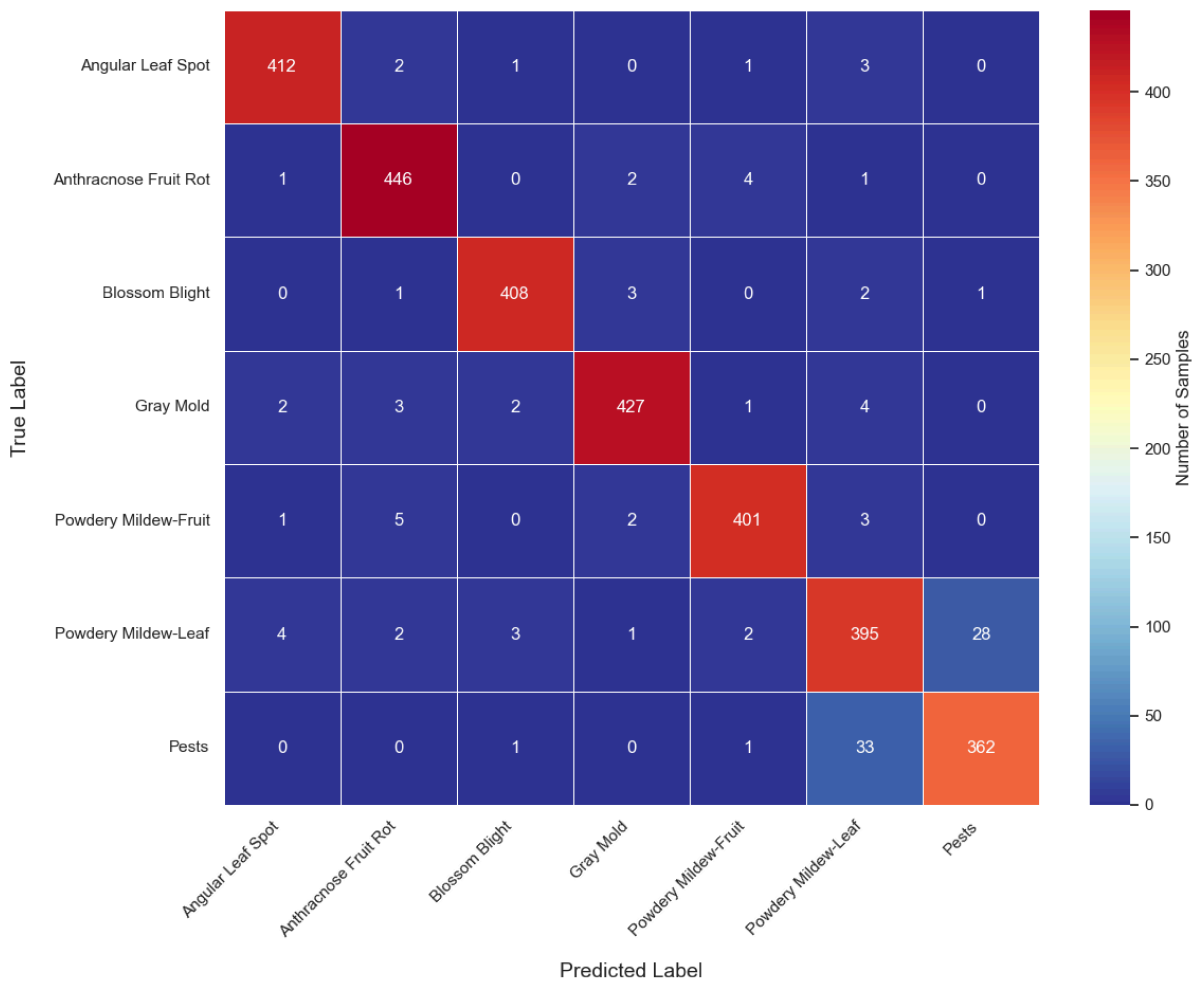
Confusion matrix heatmap of YOLOv8n-DSLW on test set (7 strawberry pest and disease categories; vertical axis = ground truth, horizontal axis = prediction; cell values represent the number of sample instances).

Experiment 5: After integrating all improved modules proposed in this paper, the model’s mAP@0.5 is boosted to 94.3%, and the F1-score reaches 0.901. Among all categories, the AP of the Anthracnose Fruit Rot category sees the most prominent improvement, rising from 0.818 to 0.958. In addition, the confusion matrix in Figure 10 visually demonstrates the core classification flaws of the model: the six disease categories achieve outstanding discrimination performance within the group, with less than five misclassified samples between any two disease types. Major classification confusion of the model occurs between Class 6 (powdery mildew leaves) and Class 7 (tiny pests). A total of 28 tiny early powdery mildew lesions are misclassified as pests, while 33 pest targets are falsely identified as powdery mildew lesions. The high similarity in grayscale textures and pixel sizes between tiny early lesions and pest bodies under leaf occlusion is the primary cause of such cross-category confusion.

Based on the existing ablation results, this study analyzes the proposed improvements from two perspectives: module-level performance gain and lightweight computational constraints. Introducing any single improvement module alone slightly increases the number of parameters and computational cost, but only brings limited accuracy improvement. In Exp1, where only the Dense+SA module is introduced, the GFLOPs decrease to 6.32, while mAP increases by only 0.5%. In Exp2, where only P2+WIoU is adopted, the GFLOPs increase to 12.37, but mAP improves by only 0.2%. In Exp3, where only LSKA is used, the GFLOPs reach 21.37, with an mAP improvement of 0.5%. The limited gain obtained from each individual module indicates that simply increasing computational complexity cannot achieve substantial accuracy improvement.

After the four modules are jointly integrated, mAP improves by 3.2%, which represents the accumulated gain produced by the synergistic and complementary effects of the four structural designs rather than by merely stacking computational complexity. When any one of the improvements is removed, the detection accuracy decreases noticeably, indicating that each module is specifically designed to address key challenges in greenhouse tiny lesion detection, including tiny disease targets, leaf occlusion, and complex background interference. Therefore, these modules provide irreplaceable scene-adaptive value rather than ineffective network expansion.

The LAMP pruning module plays a key role in constraining computational cost. The original unpruned network with all four modules contains approximately 5.32M parameters and requires about 34.9 GFLOPs. After applying the proposed LAMP layer-adaptive pruning strategy, the model is compressed to 4.386M parameters and 27.6 GFLOPs, corresponding to a 17.6% reduction in parameters and a 20.9% reduction in computational cost. The pruning strategy is specifically designed to offset the increased computational burden caused by multi-module integration and therefore serves as the core lightweight design of this study. Without LAMP optimization, the computational overhead of the model would be considerably higher than its current level.

The 84 FPS achieved by the baseline model is obtained at the expense of tiny-target feature representation. The native YOLOv8n does not include a P2 shallow detection branch and directly discards fine-grained textures after four-fold downsampling, thereby exchanging the loss of early-stage lesion features for faster inference speed. Such high-speed inference has limited practical value for early agricultural disease detection. In contrast, the proposed model achieves 45 FPS, satisfying the real-time monitoring requirement for greenhouse embedded devices, namely ≥40 FPS. This performance falls within a practical engineering deployment range and reflects a balanced trade-off between detection accuracy and deployment requirements, rather than an uncontrolled sacrifice of speed for higher accuracy metrics.

Compared with the original YOLOv8n model, the proposed YOLOv8n-DSLW model demonstrated substantially superior detection performance. Although the number of parameters increased slightly, significant improvements were achieved in disease recognition accuracy, feature extraction capability, and robustness under complex field scenarios.

**Figure 11:**
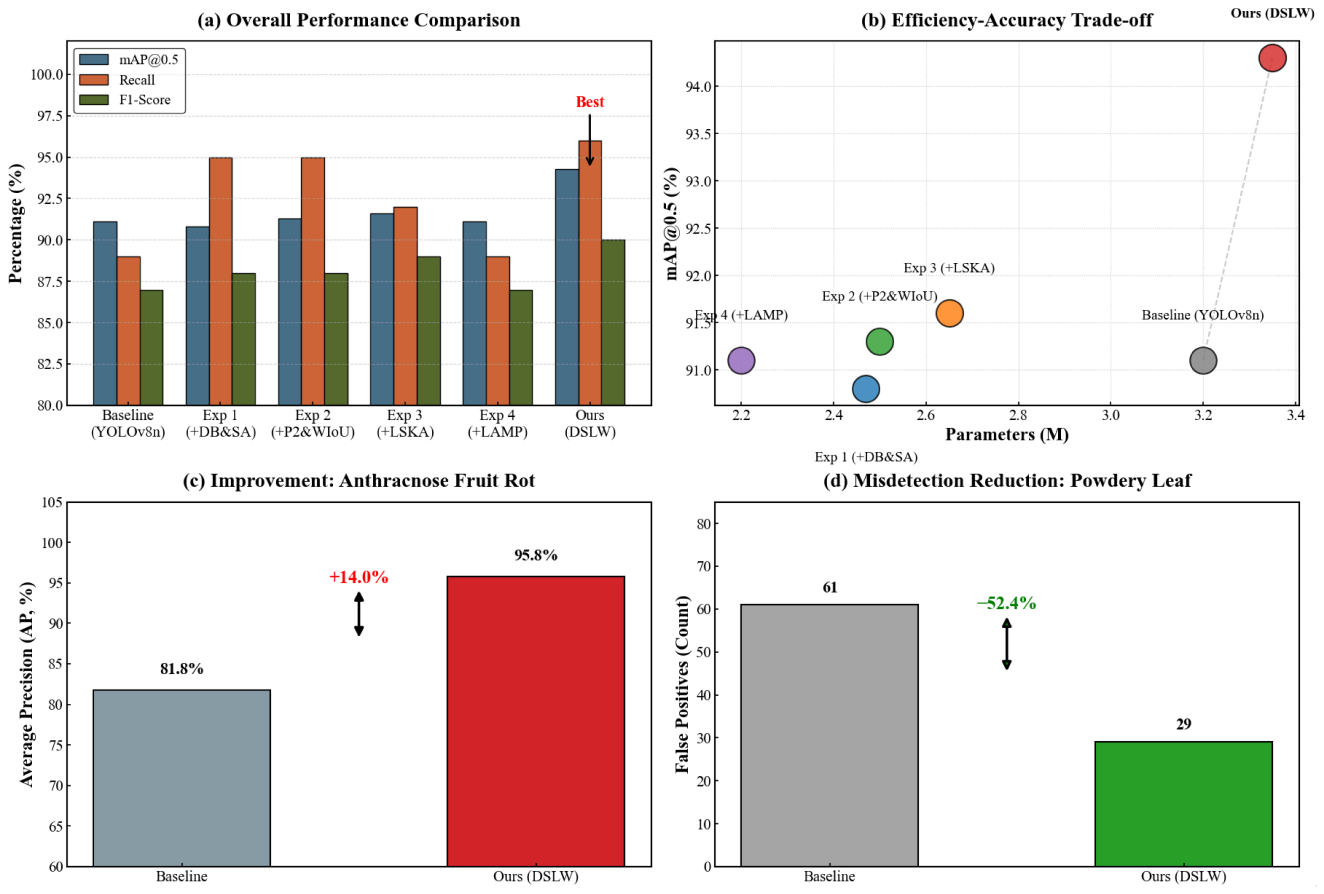
Results of Ablation Experiments.

The quantitative comparison results of the ablation experiments are shown in Figure 11. Figure 11a presents a bar-chart comparison of three key metrics, namely Recall, mAP@0.5, and F1 score, under different combinations of improvement modules. It can be observed that the performance improvement is limited when only a single module, such as Dense+Shuffle Attention, P2+WIoU, LSKA, or LAMP, is introduced. In contrast, when all four improvement modules are integrated to construct YOLOv8n-DSLW, all three metrics reach their peak values, demonstrating the synergistic enhancement effect among the proposed modules.

## 4. Discussion

The experimental results indicate that YOLOv8n-DSLW improves the visual detection of tiny strawberry diseases and pests in greenhouse images. From the perspective of greenhouse vision sensing, the proposed model addresses several practical challenges commonly observed in sensor-captured images, including weak lesion textures, complex canopy backgrounds, uneven illumination, and partial leaf occlusion. Nevertheless, several aspects should be further discussed regarding model effectiveness, dataset adaptability, deployment potential, and application limitations.

(1) Model Performance and Effectiveness of the Proposed Modules

The ablation experiments confirmed that ShrinkRDB, the P2 enhancement layer, the LSKA attention mechanism, and LAMP pruning all contributed positively to model performance improvement. The collaborative integration of these modules enabled the model to exhibit significant advantages in tiny object detection and complex greenhouse background scenarios. Among them, the P2 layer demonstrated the most significant improvement in recall for early-stage tiny lesions, whereas the LSKA module effectively reduced background false-positive detections. These results indicate that multi-scale feature enhancement and global attention modeling constitute key strategies for solving greenhouse visual detection problems in sensor-captured images [44].

(2) Dataset and Scenario Adaptability

This study employed a greenhouse image dataset containing seven disease categories under varying illumination conditions and complex backgrounds, thereby test the scenario adaptability of the proposed model. However, practical greenhouse production scenarios may involve cultivar differences, extreme environmental conditions, and mixed occurrences of diseases and pests. Therefore, future work should further expand the dataset by incorporating multiple strawberry varieties, cultivation regions, and growth stages to improve the cross-scenario robustness and stability of the model [45].

(3) Lightweight Design and Deployment Potential

The LAMP pruning strategy effectively reduced model complexity while maintaining detection accuracy, enabling YOLOv8n-DSLW to possess considerable potential for deployment-oriented greenhouse visual monitoring. The proposed model has the potential to be adapted for greenhouse inspection robots, mobile devices, and embedded platforms. Nevertheless, further lightweight optimization and hardware-specific test are still required for ultra-low-computing-power devices. Future studies may combine knowledge distillation and model quantization techniques to further compress the model.

(4) Detection Targets and Application Limitations

This study primarily focused on the visual detection of common strawberry diseases and pests in RGB greenhouse images. However, a small number of missed detections still occurred for diseases with extremely subtle early-stage symptoms and weak visual differences from healthy tissues. Moreover, functionalities such as pest dynamic tracking and disease severity quantification were not included in the current framework. In future work, temporal information and multispectral data may be incorporated to improve early diagnosis capability, while disease severity assessment modules can also be integrated into the detection framework.

(5) Field Application and Industrial Value

The proposed model can be integrated into intelligent greenhouse plant protection and camera-based monitoring systems to realize automatic disease and pest recognition, localization, early warning, and precision pesticide application [46]. Consequently, the model can support reduced pesticide usage, decreased labor costs, and improved crop yield and quality, thereby providing practical value for promoting the digital and sensor-based transformation of the strawberry industry. Future work may further conduct closed-loop greenhouse test experiments, optimize inference speed and interactive interfaces, and accelerate deployment-oriented application of the proposed technology.

(6) Deployment Limitations and Gaps from Real-World Scenarios

All experiments rely on controlled datasets. Despite covering diverse diseases and occlusions, our method has obvious deployment gaps. We used professional RGB cameras for manual close-shot sampling, while real greenhouses deploy low-cost cameras prone to blur, lens dirt and unstable light, which reduce detection accuracy. The 94.3% mAP@0.5 only reflects lab performance. Static data lacks long-term plant growth dynamics and extreme conditions like heavy haze. The hyperparameters of the LAMP pruning used in this study (20% global sparsity and an 8% pruning upper limit for shallow layers) were tuned and optimized solely on the greenhouse strawberry pest and disease dataset, leading to limitations in cross-scenario adaptability. When applied to disease recognition tasks involving other crops or samples with drastically varying scales, the fixed pruning ratio fails to achieve an optimal balance between accuracy and inference speed. Furthermore, this method adopts structured convolutional kernel pruning, which offers lower flexibility for model transfer compared with unstructured fine-grained weight pruning. When switching detection targets, the layered pruning thresholds must be re-iterated, raising the debugging costs for practical engineering deployment. This work only verifies algorithm feasibility rather than commercial maturity. Future domain adaptation and field tests will bridge lab-industry gaps.

## 5. Conclusions

This study focused on the challenging task of precise detection of tiny diseases and pests in sensor-captured greenhouse strawberry images. Aiming at lightweight architecture design, high detection accuracy, and strong robustness, the YOLOv8n model was systematically improved, and a novel AI-enabled visual detection model, termed YOLOv8n-DSLW, was constructed [47]. The main conclusions are summarized as follows.

The integration of ShrinkRDB dense connections and the Shuffle Attention mechanism effectively preserves the texture features of tiny lesions while suppressing complex greenhouse background interference. Meanwhile, the proposed strategy improves detection recall while reducing the number of model parameters.

The introduction of the high-resolution P2 detection layer combined with the WIoU dynamic regression loss significantly alleviates the missed detection problem and bounding box localization deviation of early-stage tiny lesions, thereby improving convergence stability and localization accuracy [48].

The SPPF-LSKA module enlarges the receptive field and captures global contextual information, thereby enhancing lesion feature responses. Consequently, the model maintains high detection accuracy even under complex greenhouse background conditions and severe leaf occlusion scenarios.

The LAMP strategy efficiently removes redundant parameters and achieves model lightweighting. While maintaining detection accuracy, the proposed strategy improves inference speed and enhances the adaptability of the model for deployment-oriented edge-device applications [49].

The final YOLOv8n-DSLW model achieved a mAP@0.5 of 94.3%, representing an improvement of approximately 6 percentage points compared with the original YOLOv8n model. Furthermore, the proposed model achieved the best performance among the seven categories of strawberry disease and pest detection tasks. Therefore, under the controlled experimental conditions of this study, the proposed method achieves high detection accuracy and inference speed, offering viable technical ideas and algorithmic foundations for AI-enabled health monitoring of greenhouse crops and sensor-based pest and disease management. Although the YOLOv8n-DSLW model proposed in this study achieved favorable detection performance under controlled experimental conditions, it still has clear application limitations, including the gap between the dataset imaging environment and real greenhouse monitoring scenarios, the limited scenario adaptability of pruning parameters, deficiencies in recognizing tiny targets under extreme occlusion and weak-texture conditions, and considerable fluctuations in edge-device performance caused by different hardware environments. Therefore, the proposed algorithm should be regarded only as a scenario-oriented optimization prototype for tiny strawberry disease detection, rather than a general-purpose and fully mature commercial system. Future work should further improve and optimize the model by expanding the dataset, enhancing cross-domain adaptability, and conducting long-term field validation.

## Figures and Tables

**Table 1:** Dataset Partition and Sample Independence Statistics. Note: The number of independent plant samples corresponds one-to-one with the number of raw images; duplicate samples are verified by full-dataset pHash similarity detection.

Dataset Split	Original Images	Corresponding Independent Plant Samples	Number of Duplicate Samples	Images After Augmentation
Training Set	2430	2430	0	4756
Validation Set	270	270	0	270
Test Set	300	300	0	300
Total	3000	3000	0	5326

**Table 2:** Experimental Environment Configuration.

Category	Detailed Configuration Information
Hardware Platform	Intel Core i9-10900K processor (3.70 GHz); NVIDIA GeForce RTX 3090 GPU (24 GB memory); 64 GB RAM
Operating System	Ubuntu 20.04 LTS 64-bit
Deep Learning Framework	PyTorch 1.13.0, CUDA 11.6, cuDNN 8.5.0
Development Dependencies	Python 3.8, OpenCV-Python 4.5.5, Albumentations data augmentation library
Experimental Framework	Model training and development were conducted based on the Ultralytics YOLOv8 open-source framework
Experimental Benchmark Conditions	All ablation experiments, comparative experiments, and performance evaluations were conducted under the same experimental environment to ensure consistency and fairness

**Table 3:** Training Hyperparameter Settings.

Parameter Name	Configuration Details
Optimizer	AdamW
Input Image Resolution	640 × 640
Total Training Epochs	100 Epochs
Batch Size	16
Initial Learning Rate	0.01
Learning Rate Decay Factor	0.01
Number of Data Loading Workers	8
Data Augmentation Strategy	Offline augmentation, online Mosaic augmentation, and HSV color perturbation were enabled during the first 90 epochs. Mosaic augmentation was disabled during the final 10 epochs to stabilize model convergence.
LAMP global target pruning ratio	20%
Maximum pruning sparsity limit for shallow convolutional layers	8%
Fine-tuning epochs after pruning	30 Epochs

**Table 4:** Performance Comparison With and Without Validation Set.

Evaluation Metric Value	Using Validation Set (Standard Scheme)	Without Validation Set (Control Scheme)	Absolute Difference
mAP@0.5	0.943	0.937	0.6%
mAP@0.5:0.95	0.775	0.766	0.9%
Precision	0.931	0.924	0.7%
Recall	0.882	0.875	0.7%

**Table 5:** Experimental Results of Edge Devices. Note: Peak memory refers to the system-level peak memory occupancy of the whole inference process, including model weights, intermediate feature map cache, inference framework runtime overhead, and system background memory, rather than only the file size of model weights. TX 3090 FPS is tested under the native PyTorch framework with FP32 full precision, without any deployment-level optimization. This set of data is used for fair comparison of inherent computational complexity across different models. Raspberry Pi 5 FPS is tested under ONNX Runtime with full deployment optimizations (LAMP structured pruning + INT8 full-integer quantization + Conv-BN operator fusion), which is used to verify practical edge deployment feasibility. The two sets of frame rate data have different test objectives and optimization levels, and are not suitable for direct numerical comparison. All inference tests on Raspberry Pi were repeated three times, and the average value was taken as the final result.

Model Configuration	mAP@0.5	RTX3090FPS	Raspberry Pi 5 FPS	Peak Edge-Device Memory Usage (MB)	Deployment Feasibility (≥40 FPS)
Baseline YOLOv8n (Unoptimized)	0.911	84	22.6	415	No
YOLOv8n-DSLW FP32 (Unoptimized)	0.943	45	3.9	692	No
YOLOv8n-DSLW (LAMP Pruning Only)	0.941	46	10.2	568	No
YOLOv8n-DSLW (Pruning + INT8 Quantization + Operator Fusion)	0.938	47	42.3	376	Yes

**Table 6:** Class-wise quantitative metrics of the baseline model and YOLOv8n-DSLW.

Category	YOLOv8n-DSLWAP/Prec/Recall	Baseline YOLOv8n AP/Prec./Recall	Metric Improvement
1 Angular Leafspot	93.6/92.4/87.5	90.2/86.8/80.1	+3.4 AP
2 Anthracnose Fruit Rot	95.8/94.0/90.3	81.8/84.2	+14.0 AP
3 Blossom Blight	94.1/93.2/88.1	90.7/87.5/81.5	+3.4 AP
4 Gray Mold	94.7/93.6/88.9	91.4/88.1/82.3	+3.3 AP
5 Powdery Mildew Fruit	93.2/92.1/86.7	90.1/86.3/79.6	+3.1 AP
6 Powdery Mildew Leaf	93.9/93.5/87.8	90.5/87.2/80.8	+3.4 AP
7 Tiny Pests	92.5/91.2/85.4	87.6/82.5/74.3	+4.9 AP

**Table 7:** Ablation Study Results of Different Improvement Modules. Note: All LAMP pruning experiments adopt a global sparsity of 20% and a maximum pruning constraint of 8% for shallow layers uniformly, with 30 fine-tuning epochs after pruning. LAMP refers to structured convolutional kernel pruning rather than unstructured pruning of individual weights.

Exp	Dense+SA	P2+WIoU	LSKA	LAMP	Params (M)	FLOPs (G)	Recall	mAP@0.5	FPS	F1 Score	Precision
Baseline	×	×	×	×	3.012	8.20	0.814	0.911	84	0.877	0.872
1	√	×	×	×	2.315	6.32	0.819	0.876	80	0.829	0.876
2	×	√	×	×	2.927	12.37	0.851	0.913	49	0.886	0.913
3	×	×	√	×	5.851	21.37	0.848	0.916	51	0.887	0.916
4	×	×	×	√	2.456	7.15	0.823	0.911	80	0.872	0.911
5 (Ours)	√	√	√	√	4.386	27.6	0.882	0.943	45	0.901	0.931

## Data Availability

The data presented in this study are available from the corresponding author upon reasonable request. Due to ownership and redistribution restrictions, the raw dataset is not publicly available but may be obtained from the corresponding author upon reasonable request.
